# Evaluation of the cold island effect of the urban parks in the main urban area of Wuhan from the perspective of supply and demand

**DOI:** 10.3389/fpubh.2025.1523210

**Published:** 2025-03-17

**Authors:** Jufang Song, Yongxuan Qiao, Yihan Liu

**Affiliations:** School of Urban Design, Wuhan University, Wuhan, China

**Keywords:** supply and demand evaluation, cold island effect, urban parks, planning strategy, urban heat and cold islands

## Abstract

**Background:**

Rapid urbanization has led to a series of “urban diseases” that have garnered significant social attention. Among these, the urban heat island effect has emerged as one of the most pronounced environmental concerns, presenting formidable challenges for urban planning in terms of sustainable development and environmental livability. In this process, the construction of urban parks is particularly susceptible to discrepancies between supply and demand.

**Methods:**

In this study, urban parks with an area of more than 3hm^2^ in the main urban area of Wuhan were selected as research objects. Utilizing remote sensing data and urban vector data, this study applied kernel density analysis and Thiessen polygons development to assess the supply capacity of parks’ cold islands from a supply perspective, and the residents’ cold island demand level index from a demand perspective.

**Results:**

The findings revealed that ① The spatial distribution of cold island supply and demand exhibited significant heterogeneity. High-supply units were strongly correlated with water body distribution, while high-demand units aligned closely with population density and POI density centers, displaying a “scattered overall, locally concentrated” pattern. ② A significant supply–demand mismatch in cold island effects was observed, with 19 units (accounting for approximately 40%) exhibiting insufficient supply relative to demand. These units were predominantly concentrated in areas with complex building environments, high population density, low vegetation coverage, and poor landscape connectivity.

**Discussion and conclusions:**

Drawing on these results, the study established an interplay between supply and demand perspectives by applying the theory of locational entropy and proposed optimization strategy for urban parks in Wuhan, aiming to achieve “a match between supply and demand in cold islands” across varying equilibrium stages of the research units. Specific measures include: optimizing the scale and layout of existing parks, reserving green spaces for ecological restoration, strengthening the protection of blue-green ecological foundations, and establishing a blue-green cold island corridor network to enhance ecological connectivity. Our work extends the understanding of the cold island effect of urban parks, assisting urban planners in proposing more targeted and effective management strategy and measures to improve the urban thermal environment, thereby contributing to the creation of healthy, equitable, and sustainable cities.

## 1 Introduction

In recent years, the global climate has exhibited significant fluctuations, leading to a rise in the frequency of extreme weather events, thereby posing formidable challenges to urban development and human survival. Of particular concern is the urban heat island problem, which has garnered widespread attention due to its strain on various aspects of the urban system, including urban construction space, urban ecology, and urban energy supply (1, 2). Meanwhile, the emergence of the urban heat island effect, characterized by elevated summer temperatures, has been found to be linked to increased greenhouse gas emissions, exacerbation of air pollution, and adverse effects on public health and living comfort (3, 4), which warn against sustainable urban development. As an essential part of the urban system, urban parks exhibit a noticeable cold island effect (5, 6). They play an essential role in maintaining ecosystems (7) and safeguarding residents’ physical and mental health by providing quality landscapes and activity spaces (8).

Extensive studies on the urban parks’ cold island effect home and abroad can be summarized as follows: ① Research on influencing factors from the perspective of cold island supply: Numerous studies have demonstrated that the cooling effects of parks are closely associated with their area and shape (9–11). Generally, larger parks with more compact shapes exhibit superior cooling effect. Additionally, the internal landscape composition of parks, including water bodies, green vegetation, etc., has been widely recognized as critical factors influencing cooling performance. Kong et al. highlighted that vegetation coverage is a primary factor affecting the intensity and distribution of cold islands, showing a significant positive correlation (12). However, Xie et al. found that water bodies contribute most significantly to the park cold island effect, while vegetation coverage shows no significant correlation with the average land surface temperature within parks (13). In cases where water body coverage is high, the cooling effect of vegetation may be attenuated due to the interactive influences of multiple variables on the cold island effect. Furthermore, the internal green space structure of parks also plays a role in mitigating the urban heat island effect, albeit with variations (14). Chen et al. suggested that tree-shrub-grass communities exhibit the most effective cooling performance (15), whereas Zhang et al. experimentally demonstrated that tree-grass-dominated green spaces achieve the greatest temperature reduction (16). These differences may stem from variations in vegetation types and their spatial configurations. However, the cooling effects of parks are not only influenced by their internal landscape features but also by surrounding environmental conditions such as impervious surfaces, building morphology, and composition (e.g., building density (BD) and building height (BH)) (17–19). Additionally, the cooling effects of urban parks are closely associated with the spatial aggregation of green spaces and resident visitation patterns. Research indicates that the intensity of the park cold island effect is significantly positively correlated with the average proximity of green spaces, i.e., the more concentrated the distribution of green spaces, the stronger the intensity of the cold islands (20). Simultaneously, the cooling effects are inversely proportional to the frequency and distance of park visits by residents (21). ② Research on residents’ physical and mental health from the perspective of cold island demand: Living close to recreation-friendly urban parks is conducive to improving residents’ physical fitness and self-rated health (22). Residents’ frequent use of parks can effectively reduce their risk of cardiovascular disease (23). Additionally, the park’s vegetation environment has a positive impact on people’s emotional recovery (24). Above all, existing research only discusses the influencing factors of the cold island effect in urban parks or explores the positive benefits on people’s physical and mental health. However, there are very few studies on alleviating the heat island effect from the perspective of urban cold and heat island supply–demand equilibrium. There is also a lack of research on park planning in spatial decision-making that considers human needs and insufficient comprehensive quantitative research on the integration of “demand subjects” (residents) and “supply subjects” (urban parks). This deficiency makes it challenging to formulate comprehensive solutions to address urban heat environment issues.

The relationship between supply and demand generally refers to the dynamic and relative relationship between production and consumption in the commodity market economy. Correspondingly, at the level of urban park systems, it denotes the equilibrium pursued between the cold island supply capacity of urban parks and the targeted needs of residents for living and production. In the context of high-quality development and the awakening of national health awareness, it is of significant theoretical and practical importance to quantitatively analyze the cool island effect of urban parks from the perspective of supply and demand in order to plan and utilize it reasonably to maximize its ecological service functions. This is crucial for mitigating the urban heat environment and promoting the construction of ecologically livable cities (25).

As a prototypical “furnace city,” Wuhan has faced increasingly prominent thermal environment problem issues in recent years due to the city’s rapid expansion and the sharp growth of its population. Therefore, this study focuses on the main urban area of Wuhan, where the urban heat island (UHI) effect is most pronounced. By integrating multi-source data and establishing a dual-perspective evaluation system from both supply and demand sides, the research aims to ① reveal the spatial distribution characteristics and patterns of the urban parks’ cold island effect from both supply and demand perspectives; ② analyze the degree of supply–demand matching for the cold islands of the urban parks and their spatial differentiation patterns; ③ propose targeted park planning optimization strategies based on the supply–demand matching results, providing scientific and reasonable suggestions to the construction sequence and optimization emphasis of the urban parks in a point-to-point way.

## 2 Materials and methods

### 2.1 Description of the study area

**Figure 1:**
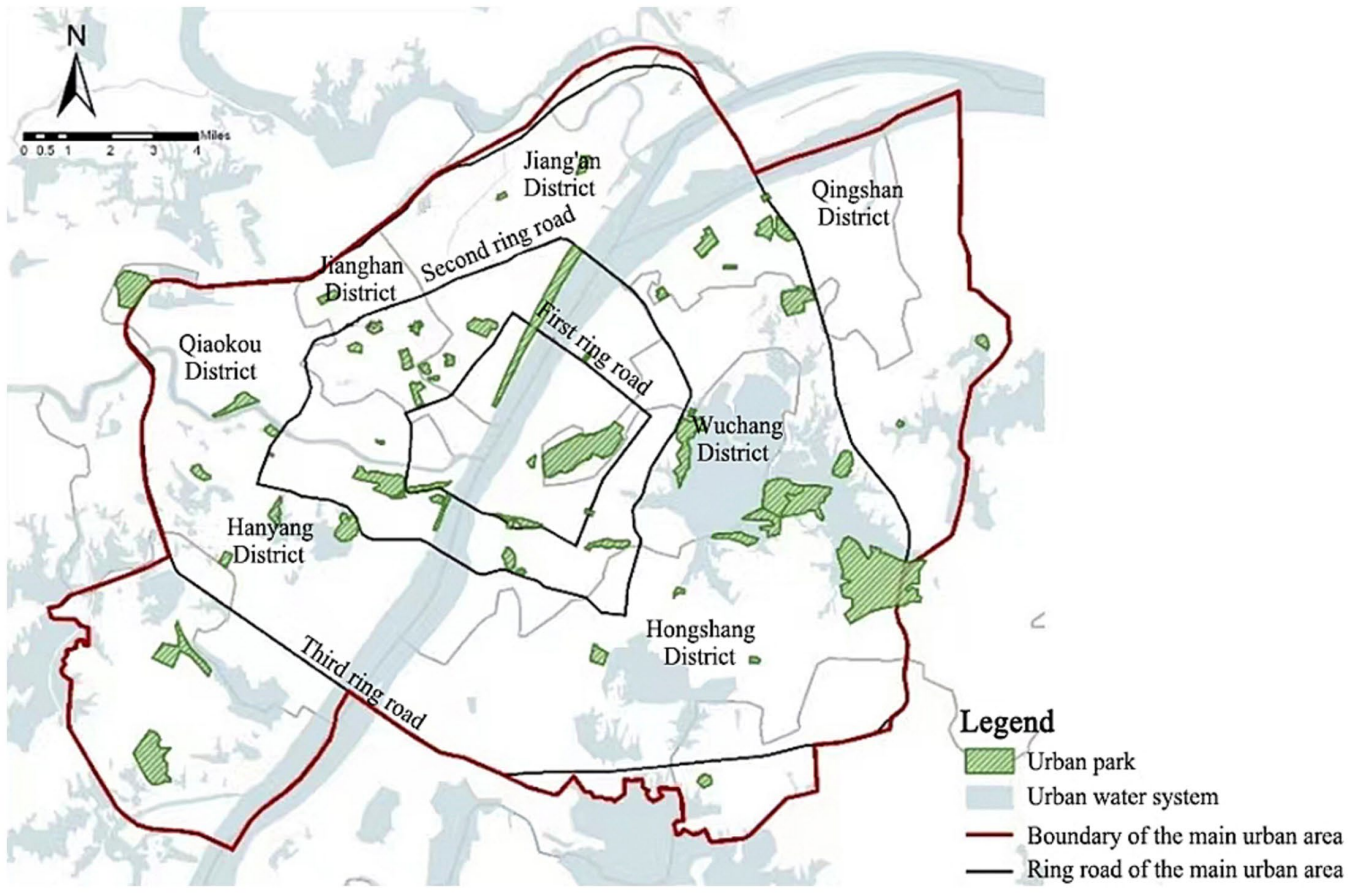
Distribution of the urban parks with an area of more than 3hm^2^ in the main urban area of Wuhan.

Situated in the eastern part of the Jianghan Plain, Wuhan (29°58′ ~ 31°22′N, 113°41′ ~ 115°05′E) is at the confluence of the Yangtze River and Han River. It serves as the capital city of Hubei Province and the core city of the city cluster in the middle reaches of the Yangtze River. The city covers an area of 8,494 km^2^ and includes 13 municipal districts, among which Jiang’an District, Jianghan District, Qiaokou District, Hanyang District, Wuchang District, Hongshan District, and Qingshan District are the main urban areas, with an area of 678km^2^. Characterized by a subtropical monsoon climate, Wuhan experiences an annual average temperature ranging from 15.8 to 17.5°C, with summer extreme temperatures reaching up to 41.3°C (26), creating significant thermal environment challenges. This paper selected 60 urban parks with an area of more than 3hm^2^ in the above-mentioned seven main urban areas as the research objects to conduct the supply and demand evaluation of the urban parks’ cold island effect in the main urban area of Wuhan (Figure 1).

### 2.2 Data sources and processing

The remote sensing data source in this paper is the Landsat 8 satellite image on 29 July 2018 with orbit number 123/39, obtained from the official website of the United States Geological Survey (USGS).^1^ The reasons for choosing this image are as follows: ① On this day, the maximum temperature reached 37°C, significantly intensifying the urban heat island effect and increasing residents’ demand for the cooling effects of urban parks. This provides a clearer reflection of the supply–demand relationship of urban parks’ cold island effects, particularly in identifying areas with supply–demand imbalances. ② The Landsat image for this day was cloud-free and of high quality, ensuring reliable analysis of land surface temperature and other parameters. ③ Studies show that under breezy conditions with little prior precipitation, air and land surface temperatures correlate more closely. Wuhan had no rainfall for 10 consecutive days prior to July 29, 2018, with a wind speed of force 2 (breezy) recorded on that day, further validating the suitability of this date for reliable thermal analysis.

The image data were processed through the remote sensing image processing software platform ENVI and the GIS (Geographic Information System) software platform ArcGIS 10.3. The urban vector data included the road network data of the main urban area of Wuhan,^2^ POI (Points of Interest) data,^3^ Chinese population spatial distribution raster data,^4^ and urban park data obtained from information released by Wuhan Municipal Landscape Gardens and Forestry Bureau, and 2018 Wuhan Statistical Yearbook and corresponding socio-economic statistics and so on.

### 2.3 Analytical workflow

**Figure 2:**
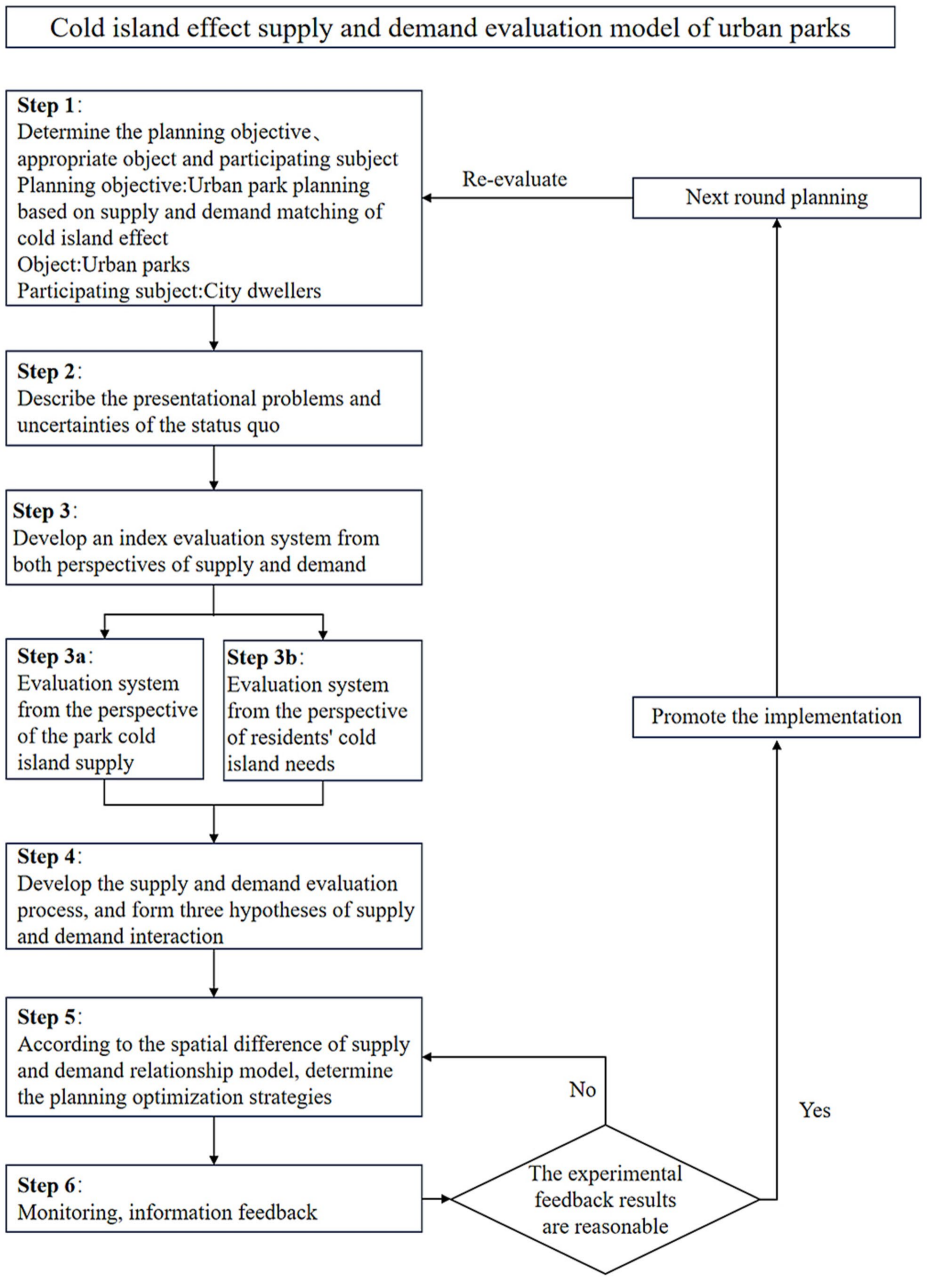
Logic diagram of the supply and demand evaluation model of the urban parks’ cold island effect.

Figure 2 illustrates the operational process of the model for evaluating the cold island effect of the urban parks from the perspective of supply and demand:① define the objectives and targets of the planning; ② conduct a comprehensive assessment of the current situation and problems by taking into account the resource base and regional status of the parks; ③ develop an index system from both the supply and demand perspectives; ④ formulate the evaluation process to presuppose the relationship between supply and demand; ⑤ determine optimization strategies for different supply–demand patterns; ⑥ plan for implementation and recycle this route for the next planning stage.

### 2.4 Delineation of park service units

**Figure 3:**
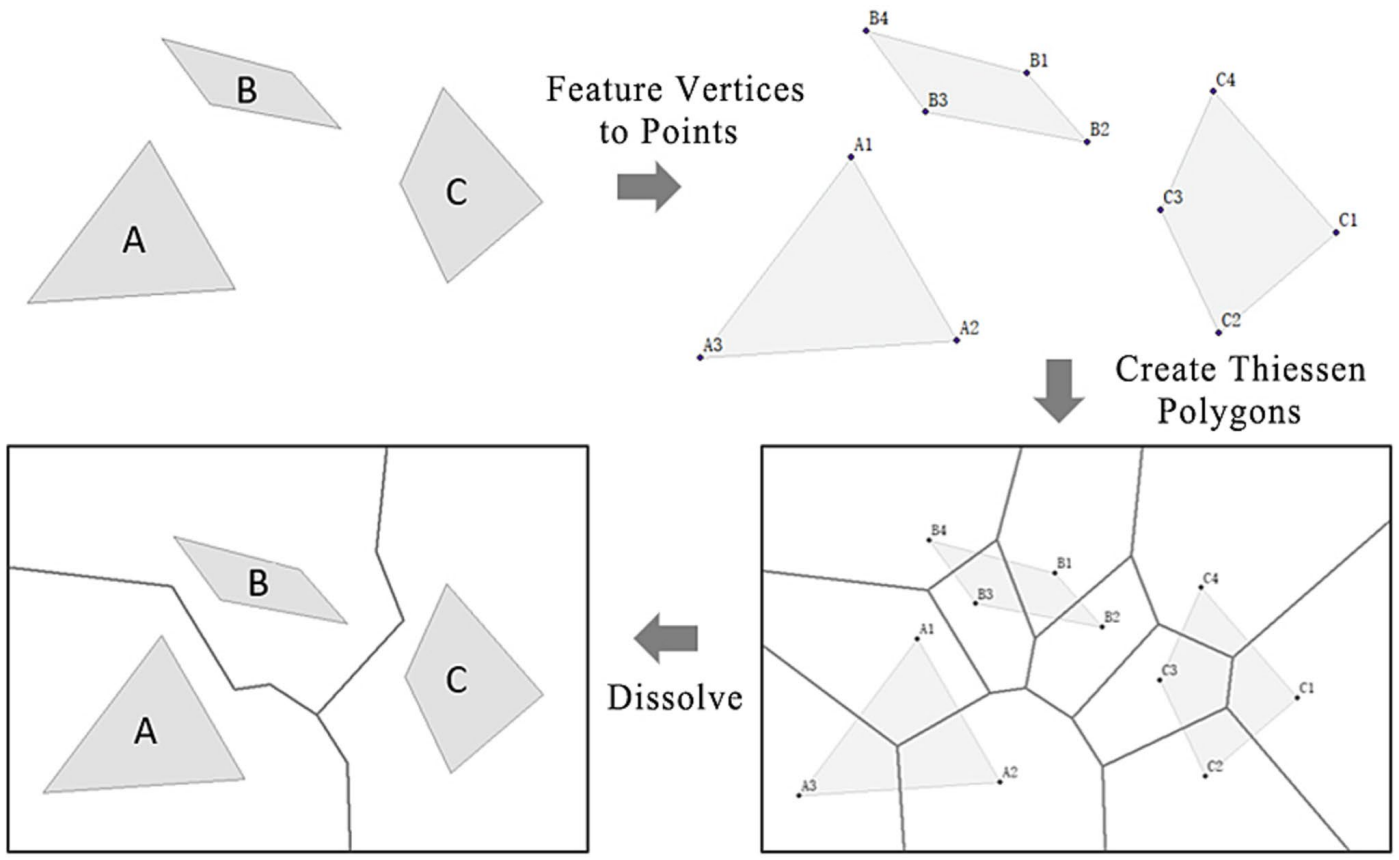
Process of generating Thiessen polygons from polygon elements.

Traditional cold island effect measurements often rely on buffer analysis, but this method has two main limitations: first, it requires predefined buffer distances, leading to subjective results; second, it struggles to accurately reflect spatial competition between parks, particularly in complex urban environments with irregular park distributions. In contrast, Thiessen polygons automatically assign available space to the nearest point element based on spatial distance, without predefined parameters. It not only removes subjectivity but also more objectively reflects spatial competition between elements. Therefore, this study divided the city into several park cold island service areas centered on urban parks by constructing Thiessen polygons. Using these service areas as spatial units, the supply and demand of the urban parks’ cold island effect were evaluated and identified. Since the city park is a polygon element, the procedure involves several steps (Figure 3). The first step is to use the “Feature Vertices to Points” tool to convert the vertices of the polygon elements into point elements. Subsequently, the “Create Thiessen Polygons” tool is utilized, followed by the “Dissolve” tool to merge the Thiessen polygons with common fields, thereby accomplishing the creation of Thiessen polygons for polygon elements.

However, the urban spatial texture is complicated, thus lacking correspondence with the urban space in actual urban research. Hence, when delineating the park service units, the Thiessen polygons of geometrical significance was combined with the regulatory planning units taking into account the spatial fabric of the city to enhance the accuracy of research.

### 2.5 Evaluation model for park cold island supply

#### 2.5.1 Evaluation index system construction from the supply perspective

The park cold island evaluation index system from the supply perspective mainly focuses on: ecological supply and social supply (Table 1). Among them, the core carrier of ecological supply is blue-green spaces, which refer to a spatial system composed of various types of water bodies, wetlands, green spaces, and other open areas (27, 28). As an essential component of urban ecosystems, blue-green spaces not only provide critical ecological services to urban residents but also significantly reduce local temperatures and create cold island effects through mechanisms such as evapotranspiration, shading effects, and water body cooling (29–31). These mechanisms play a key role in regulating urban climate and mitigating thermal environments (28). Based on this, this study focuses on three types of blue-green spaces with significant cold island effects: the park itself, vegetation, and water bodies (32, 33). Their scale and quality are crucial indicators for measuring the supply capacity of park cold islands (34, 35). Social supply can be further subdivided into access methods and per capita availability. There are two ways in which the cold island effect of urban parks is transmitted to the users, one is directly through the air, and the other requires the users to arrive within the radiation range of the park’s cold island. Given the limited spatial extent of urban parks, the per capita park area accessible to residents within its service range and the park’s accessibility have become significant factors that cannot be ignored when evaluating the supply capacity of the cool island effect (36).

**Table 1:** Evaluation index system of the park cold island effect from the supply perspective.

Supply categorization	Functional carrier	Evaluation index	Abbreviation/Unit	Definition
Ecological supply	The park itself	Park size	parkS/hm^2^	Area of urban parks in cities that can effectively provide the cold island effect
	Water body	Water size in the park	wS/hm^2^	Water area in city parks
	Vegetation cover	Fractional vegetation coverage	FVC/ -	Average of all FVC measurement points in city parks
Social supply	Mode of visit	Accessibility	A/(m^2^/person)	Residents’ accessibility to parks determined by the Gaussian two-step floating catchment area method
	Per capita access to parks	Per capita park size	perS/(m^2^/person)	Ratio of urban park area to total population in the corresponding service area

#### 2.5.2 Calculation of evaluation indicators from the supply perspective

##### 2.5.2.1 Fractional vegetation cover

The downloaded Landsat 8 series images were loaded into ENVI (The Environment for Visualizing Images) for initial processing, including radiometric calibration, FLAASH atmospheric correction, image fusion, stitching, and cropping. The fractional vegetation cover (FVC) of the study area was calculated using the pixel dichotomy model. This model assumes that each pixel’s surface consists of two components: vegetated and non-vegetated areas. The FVC value for a pixel is defined as the proportion of its area covered by vegetation. The formula is as follows:


(1) $$FVC=\frac{\text{NDVI}-NDVI_{s}}{NDVI_{v}-NDVI_{s}},$$

In Equation 1, $NDVI_{s}$ represents the NDVI value of bare soil pixels, and $NDVI_{v}$ represents the NDVI value of pure vegetation pixels. The upper and lower thresholds of NDVI were selected at a 5% confidence level, and the NDVI values within the threshold range were averaged to obtain $NDVI_{s}$ and $NDVI_{v}$.

##### 2.5.2.2 Accessibility

The two-step floating catchment area method (2SFCA) was applied to calculate the accessibility within the study area, with city parks as the supply side and residents of residential quarters as the demand side. The traditional 2SFCA ignores the importance of distance --the urban parks’ cold island supply capacity weakens as users move further away from them (37). Consequently, a Gaussian function is introduced as the distance attenuation function for 2SFCA. The Gaussian two-step floating catchment area approach (Ga2SFCA) incorporates distance as a factor in its reachability calculation, hence enhancing the realism of the results (38). The search radius $d_{0}$ calculated in this study based on the 15-min living circle definition of accessibility was 1 km. The formulas are as follows:


(2) $$R_{j}=\frac{S_{j}}{\sum_{i}^{m}D_{i}\times G\left(d_{ij}\right)},$$

(3) $$G\left(d_{ij}\right)=\frac{e^{-\frac{1}{2}\times {\left(\frac{d_{ij}}{d_{0}}\right)}^{2}}-e^{-\frac{1}{2}}}{1-e^{-\frac{1}{2}}},d_{ij}\leq d_{0},$$

(4) $$A_{i}=\sum_{j}^{n}R_{j}\times G\left(d_{ij}\right),$$

In Equation 2, $R_{j}$ is the per capita park size (m^2^/person) for the potential demanders within the search range $d_{0}$ of the urban park; i is the residential quarters within the search radius $d_{0}$; j is the city park; m is the number of the residential quarters within the search distance $d_{0}$; $D_{i}$ is the number of the inhabitants in the residential quarter i (person); $d_{ij}$ is the distance between the centroid of the residential quarter i and the boundary of the city park j; $S_{j}$ is the size of the park j (m^2^).

Equation 3 is the distance decay function – the Gaussian function.

In Equation 4, $A_{i}$ is the accessibility of the residential quarter i (m^2^/person); n is the number of the urban parks j within the search radius $d_{0}$; $R_{j}$ is the per capita park size of the demander within $d_{0}$ as required by Equation 2.

##### 2.5.2.3 Per capita park size

The number of people in demand within the park service unit is filled, i.e., the number of people living within the park’s service area is summed up and used as the basis for calculating the per capita area of the corresponding park, to measure the per capita provisioning capacity allocated to the park in the spatial layout planning process. The formula for this calculation is as follows:


(5) $$\text{per}S_{k}=\frac{S_{j}}{\sum_{t}^{e}P_{t}},$$

In Equation 5, k is the urban park service unit k; $\text{per}S_{k}$ is the per capita park size within the park service unit k; j is the urban park within the service unit k; $S_{j}$ is the size of the park j (m^2^); t is the residential quarter within the park service unit k; e is the total number of the residential quarters within the park service unit k; and $P_{t}$ is the number of the inhabitants in the residential quarter t (person).

#### 2.5.3 Park cold island supply index calculation

The comprehensive cold island supply index was determined based on six key factors: the dominant park area within the service unit (parkS), vegetation cover within the park (FVC), water area (wS), per capita park size within the service unit (perS), accessibility (A) and each index’s weight calculated using the subjective-objective composite weighting method. To enhance the accuracy of the weighting process, this study employed a combined weighting method that integrates the subjective Analytic Hierarchy Process (AHP) and the objective entropy weight method, thereby addressing the limitations of relying on a single weighting approach. Specifically, the AHP method was used to calculate subjective weights, while the entropy weight method was applied to determine objective weights. The final combined weights were derived based on the principle of minimum information entropy (39). Based on the weights and scores of the five indicators (Table 2), the park cold island supply index was obtained by weighted summation in Arcgis10.3 using the raster calculation tool. The calculation formulas are as follows:


(6) $$SW_{n}=\frac{\sqrt{SW_{\alpha n}*SW_{\beta n}}}{\sum_{n-1}^{5}\sqrt{SW_{\alpha n}*SW_{\beta n}}}$$

(7) $$SI_{k}=\sum_{n=1}^{m}S_{n}\times SW_{n},\left(n=1,2,\;\ldots \ldots ,\;5;k=1,2,\ldots \ldots ,48\right)$$

**Table 2:** Combined subjective and objective weighs from the supply perspective.

Weight type	Park size (parkS)	Water size in the park (wS)	Fractional vegetation cover (FVC)	Per capita park size (perS)	Accessibility (A)
Subjective weighs	0.29	0.17	0.32	0.08	0.11
Objective weighs	0.10	0.14	0.27	0.12	0.15
Subject-objective combination	0.18	0.17	0.31	0.10	0.14

In Equations 6 and 7, $SW_{n}$ is the comprehensive weight of the nth supply indicator, $SW_{\alpha n}$ denotes its subjective weight, and $SW_{\beta n}$ signifies its objective weight; *SI_k_* is the park cold island supply index of the unit k, and there are 48 park service units, numbered from 1 to 48; $S_{n}$ is the nth park cold island supply capacity index; n is a natural number from 1 to 5.

### 2.6 Evaluation model for residents’ cold island demand

#### 2.6.1 Construction of evaluation index system from the demand perspective

The construction of the park cold island evaluation index system (Table 3) from the demand perspective is based on the multi-level needs of urban residents, specifically including the following two aspects: ① At the physiological demand level, the focus lies on residents’ physiological health conditions and their correlation with the thermal environment. With the frequent occurrence of global high-temperature weather, urban residents face an increased risk of heat-related illnesses, cardiovascular and cerebrovascular diseases, respiratory diseases, and other health hazards (40, 41). Research has demonstrated that land surface temperature (LST) directly influences human thermal comfort and serves as a critical indicator of the urban thermal environment (42), while the spatial extent of heat island patches identifies the scale of impacted areas and pinpoints regions requiring targeted mitigation efforts (43). Therefore, land surface temperature and heat island patch size have been selected as key indicators to intuitively reveal the presence and distribution of thermal environmental risks. The higher the thermal environmental risk, the more urgent residents’ demand for park cold islands becomes. ② The psychological needs dimension focuses both on the potential impact of the hot environment on residents’ mental health and on the environmental justice issues. Studies have shown that prolonged exposure to hot environments may lead to psychological stress (44) and negative emotions among residents, which in turn reduces their life satisfaction (45). At the same time, it is emphasized that every individual should enjoy equal access to high-quality park cold island services (46, 47). The essence of spatial equity in urban parks lies in the rational allocation of space, and although the total amount of park cold island services is determined by the supply side, the quality of cold island services available per capita is influenced by the number of demanders on the demand side. For this reason, population density and POI density are introduced as key indicators for assessing exposure. Among them, the density of resident population reflects the local static demand dominated by the residential function (48); while POI (Points of Interest), as an important characterization of the functional nodes of the city, the degree of aggregation directly reflects the intensity of human activities, i.e., the nonlocal dynamic demand (49, 50).

**Table 3:** Evaluation index system of the park cold island effect from the demand perspective.

Demand categorization	Evaluation index	Abbreviation/Unit	Definition
Physiological requirement	Heat island patch size	hiS/hm^2^	Area of clustered heat island patches
	Land surface temperature	LST/°C	Land surface temperature from remote sensing image inversion
Mental requirement			
	Population density	PD/ (person/ km^2^)	Residential population per square kilometer
	POI kernel density	PoiKD/−	The agglomeration of POI (Points of Interest) in urban spatial distribution

#### 2.6.2 Calculation of evaluation indicators from the demand perspective

##### 2.6.2.1 Population density

Regions with high population density suggest a more extensive and intensive number of users, implying a higher demand for cold islands. Through the population raster data obtained from WorldPop, the population density distribution of the computational units within the study area was acquired with the following formula.


(8) $$PD_{k}=\frac{P_{k}}{S_{k}},$$

In Equation 8, k is the urban park service unit k; $PD_{k}$ is the per capita park size of the park service unit k; $S_{k}$ is the size of the park service unit k (km^2^); and $P_{k}$ is the total number of people living in the service unit k (person).

##### 2.6.2.2 POI kernel density

The kernel density of POI (Points of Interest) can characterize urban social activity levels. Regions with high POI kernel density suggest that residents engage in social activities more frequently and actively there, implying a higher demand index. Initially, the captured POI data underwent a cleaning and filtering process. Subsequently, the data coordinates were characterized as the GCS_WGS_1984 coordinate system and projected for the subsequent calculation and analysis. Then, the “Kernel Density” tool within the ArcGIS software was employed to calculate the kernel density of points of interest. The calculation of the kernel density of points in space is given in Equation 9:


(9) $$f_{n}\left(x\right)=\frac{1}{nR}\sum_{i=1}^{n}K\left(\frac{x-x_{i}}{R}\right),$$

In Equation 9, n is the number of point samples; R is the radius of the search window; $K\left(\frac{x-x_{i}}{R}\right)$ is the kernel function.

##### 2.6.2.3 Land surface temperature

Evidently, LST (Land Surface Temperature) is an essential index in the study of urban heat island effect. Especially with the gradual application of remote sensing data, various algorithms, including split-window, single-channel, and single-window algorithms, have become the most commonly used LST retrieval algorithms (51). Compared with the traditional radiative transfer equation, the single-channel algorithm has several benefits including a straightforward model, enhanced computational efficiency, and fewer required parameters. Hence, this paper chose the single-channel algorithm to calculate the land surface temperature using the following equations, ultimately yielding the land surface temperature (LST) raster image:


(10) τ=ε×τ,

(11) $$D=\left(1-\varepsilon \right)\left[1+\left(1-\varepsilon \right)\tau \right],$$

(12) $$LST=\frac{a\left(1-C-D\right)+\left[b\left(1-C-D\right)+C+D\right]\times T_{b}+D\times Ta}{C},$$

In Equations 10–12, ε is the land surface emissivity; τ is the atmospheric transmittance and can be queried on the NASA official website for the corresponding region; C and D can be calculated.

##### 2.6.2.4 Heat island patch size

The mean-standard deviation method was applied to classify the surface temperature obtained from the inversion (Table 4). Then, the heat island patches were extracted and subsequently quantified in terms of their respective actual sizes with the following formula. Due to the difference in patch sizes and the discontinuous nature of these values, the assignment of values for the real size of heat island patches was conducted to ensure the validity of data calculations. The specific assignment criteria are shown in Table 5.


(13) $$hiS_{k}=\sum_{h}^{a}I_{h},$$

**Table 4:** Classification of the land surface temperature.

Rank	Cold Island Zone	Central Temperature Zone	Heat Island Zone
Land surface temperature range T (°C)	23.5 < T ≤ 31.2	31.2 < T ≤ 35.5	35.5 < T ≤ 56.1

**Table 5:** Criteria for assigning heat island patch size.

Score I	0	2	4	6	8	10
Actual size of heat island patch h (hm^2^)	S = 0	0<S ≤ 5	5<S ≤ 10	10<S ≤ 50	50<S ≤ 100	S>100

In Equation 13, $hiS_{k}$ is the heat island patch score for the park service unit k; k is the urban park service unit k; h is the heat island patch within the service unit k; $I_{h}$ is the size-assigned score for the heat island patch h; and a is the total number of the heat island patches within the service unit k.

#### 2.6.3 Residential cold island demand index calculation

Following the same method as described in section 2.5.3, the park cool island demand index was calculated using the following formula, based on the weights and scores of the four demand indicators (Table 6).


(14) $$DW_{n}=\frac{\sqrt{DW_{\alpha n}*DW_{\beta n}}}{\sum_{n-1}^{4}\sqrt{DW_{\alpha n}*DW_{\beta n}}}$$

(15) $$DIk=\sum_{n=1}^{m}D_{n}\times DW_{n},\left(n=1,2,\;\ldots \ldots ,\;4;k=1,2,\ldots \ldots ,48\right)$$

**Table 6:** Combined subjective and objective weighs from the demand perspective.

Weight type	Land surface temperature (LST)	Heat island patch size(hiS)	POI kernel density (PoiKD)	Population density (PD)
Subjective weights	0.48	0.29	0.08	0.15
Objective weights	0.24	0.26	0.24	0.26
Subject-objective combination	0.35	0.29	0.15	0.21

In Equations 14 and 15, $DW_{n}$ is the comprehensive weight of the nth demand indicator, $DW_{\alpha n}$ denotes its subjective weight, and $DW_{\beta n}$ signifies its objective weight; $DI_{k}$ is the residential cold island demand index of the unit k, and there are 48 park service units, numbered from 1 to 48; $D_{n}$ is the nth residential cold island demand index; n is a natural number from 1 to 4.

### 2.7 Identification and evaluation of supply and demand balance relationship of cold islands in urban parks

The interaction between supply and demand perspectives means the superposition, differentiation, and comparison of supply capacity and demand targets in the regional space. To achieve this, this paper introduces the location entropy used to measure and evaluate the state of spatial distribution characteristics of factors within a given region, as a path to establish the relationship:


(16) $$LQ_{k}=\left(SI_{k}/DI_{k}\right)/\left(SI/DI\right),$$

In Equation 16, $SI_{k}$ is the park cold island supply index for the region k, $DI_{k}$ is the residents’ cold island demand index for the region k, SI is the average supply index for the cold island effect within the study area, and DI is the average demand index for the cold island effect within the study area.

The values of $LQ_{k}$ are discussed in terms of mean-standard deviation (A is the mean value of $LQ_{k}$ in the study area, while sd is the standard deviation of $LQ_{k}$), from which three kinds of relative relationships between supply and demand can be obtained:

If the value of $LQ_{k}$ is less than A-0.5sd, it indicates an unsatisfied demand in the region, implying that the supply is insufficient compared to the demand. Conversely, if $LQ_{k}$ exceeds A + 0.5sd, it suggests a relatively sufficient supply in the region, indicating that the supply exceeds the demand. In the case where $LQ_{k}$ falls within the range of A-0.5sd to A + 0.5sd, it signifies the supply and demand capacity ratio of the cold island effect in the region is within a specific range, meaning a state of “relative balance.”

## 3 Results

### 3.1 Delineation of park service units

**Figure 4:**
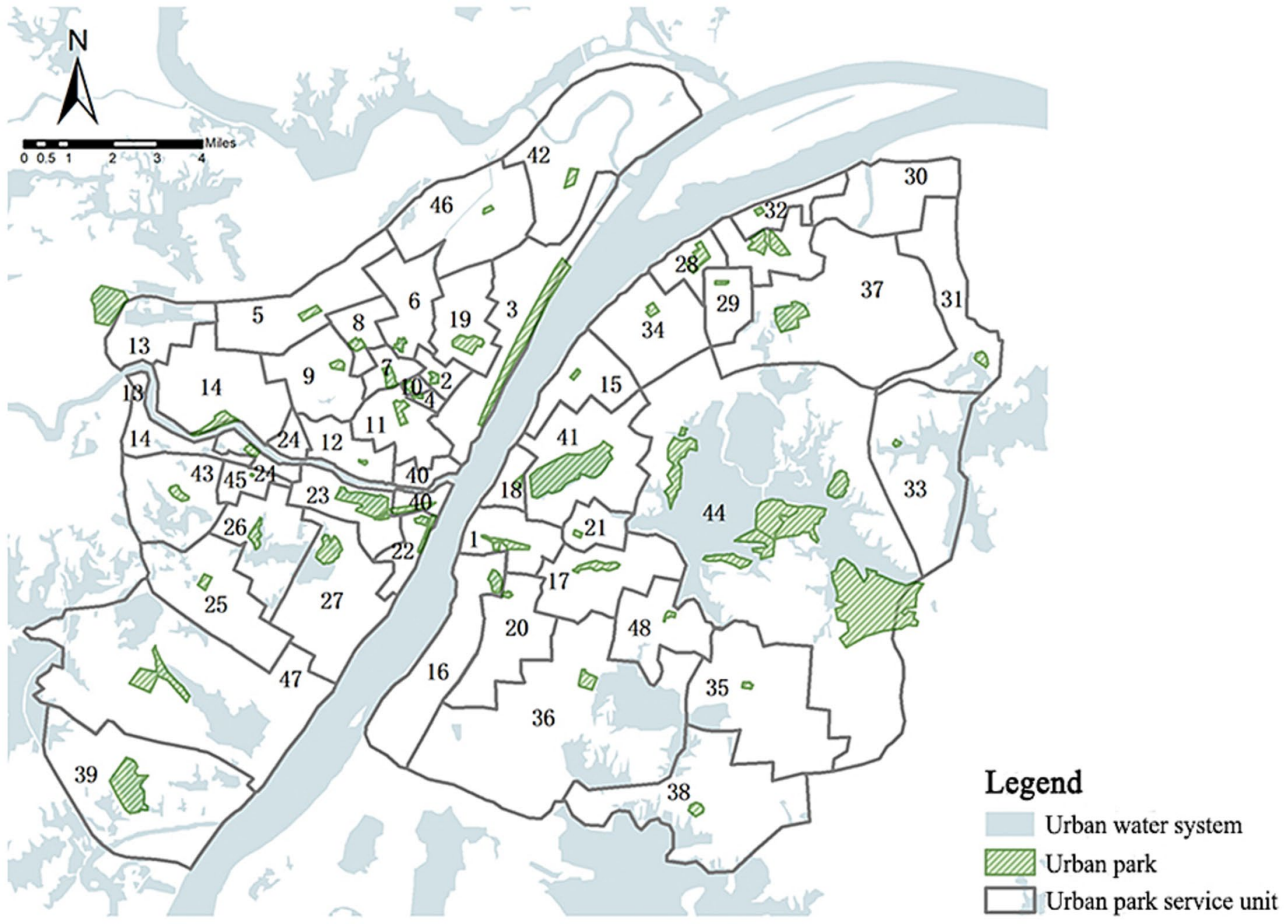
Spatial distribution of the urban park service units in the main urban area of Wuhan; The numbers 1–48 represent the park service unit number.

According to the method of 2.4, the 60 urban parks in the main urban area of Wuhan got 48 park service units. The spatial distribution map of these park service units (Figure 4) reveals that the size of service units gradually increases from the core of the study area (within the first ring road) to its outskirts (the third ring road). The urban parks in the central area along the riverbanks were mostly embedded in the urban space in the way of “small areas with multiple dots,” resulting in smaller overall service units. In the suburbs, larger patches of parkland existed, yet there were also instances where the area of the service unit was large despite the dominant urban park within it being very small, such as in the Guanshan Unit, South Lake Unit, Yunhu Unit, and Houhu Unit. The information for park service units can be queried from Supplementary Table 1.

### 3.2 Evaluation of the park cold island supply capacity from the supply perspective

#### 3.2.1 Visual analysis of park cold island supply

##### 3.2.1.1 Fractional vegetation cover

**Figure 5:**
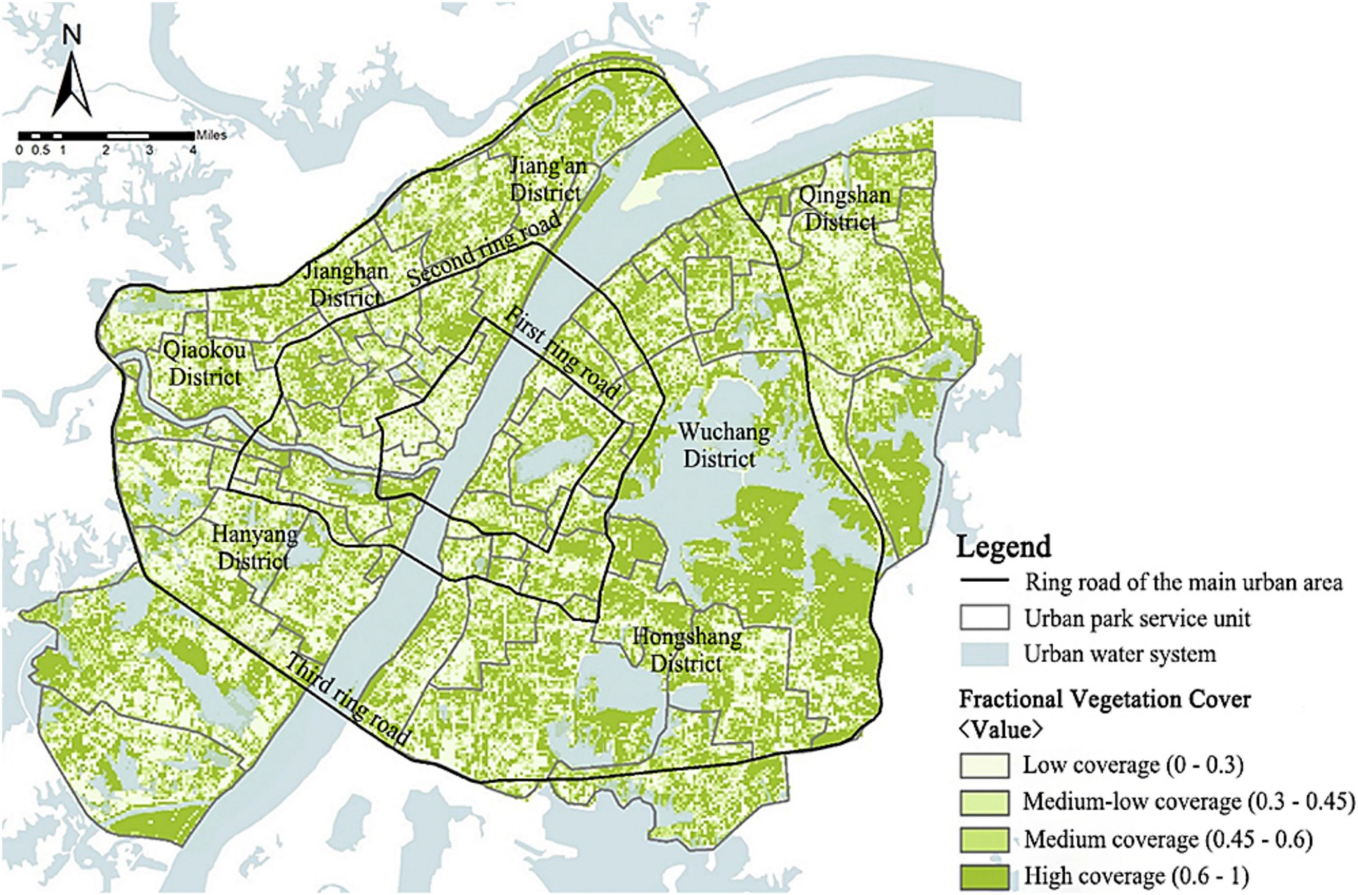
Spatial distribution of the vegetation coverage in the main urban area of Wuhan.

The raster image of vegetation cover in the study area (Figure 5) shows that the region’s global average vegetation coverage is 0.44, with a general gradual increase in the vegetation coverage from the center to the periphery, which is significantly higher on the eastern side of the study area than in the rest of the region. Several water bodies in the main urban area were with high vegetation coverage, and the “blue space” and “green space” could be effectively connected through vegetation. The East Lake Scenic Area was the area with the most extensive area of high vegetation coverage in the main urban area, while Hankou River Beach Park, Turtle Mountain Scenic Area, etc., all had a discernible concentration of clusters with high vegetation coverage.

##### 3.2.1.2 Accessibility

**Figure 6:**
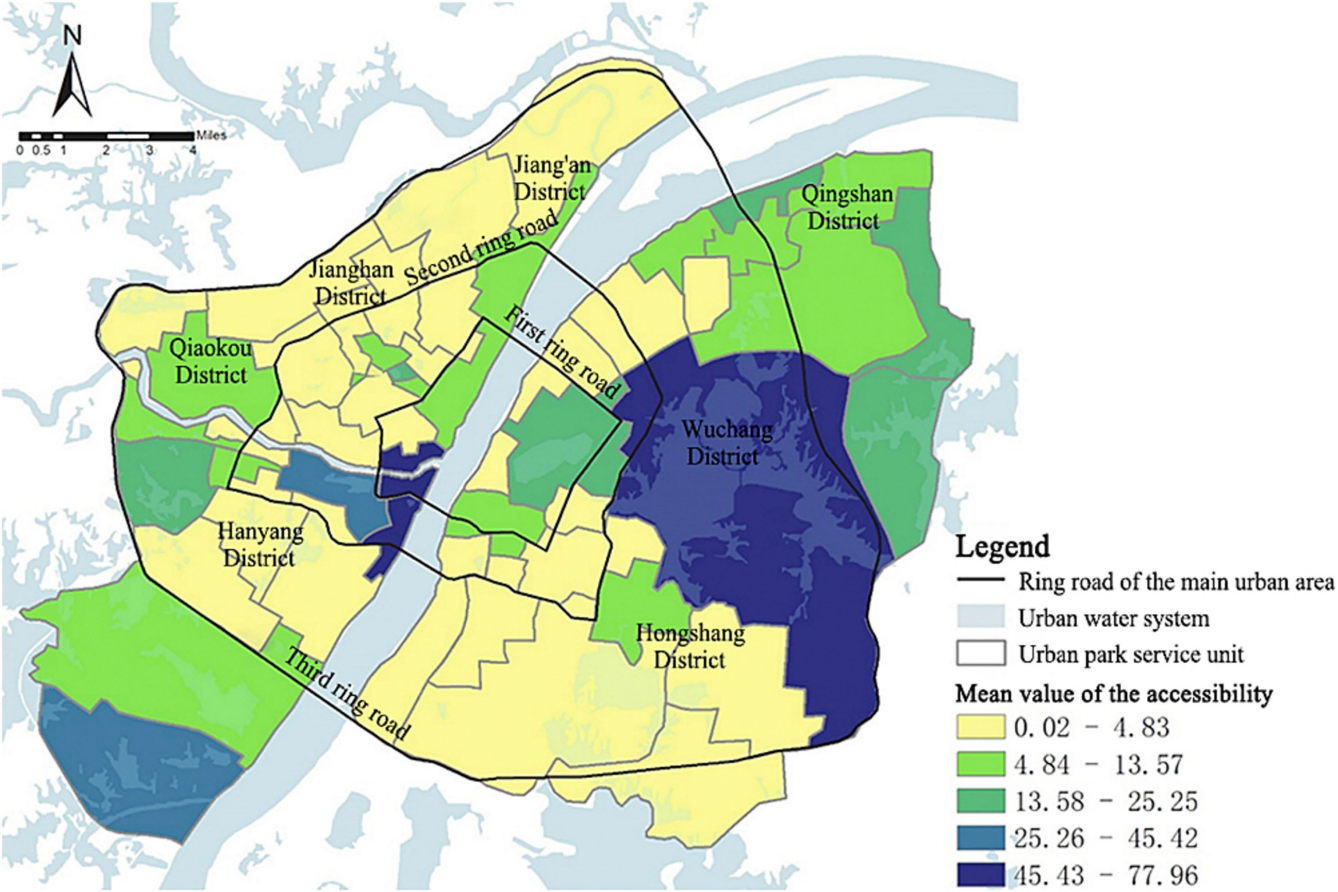
Spatial distribution of the park accessibility in the main urban area of Wuhan.

The accessibility distribution map of each park service unit (Figure 6) shows that, in general, the accessibility in the eastern region of the Yangtze River is significantly better than the western region. According to the “Series of Standards for National Garden Cities” (after this referred to as “the Standards”), the basic indicator for per capita public green space in Wuhan is 7.5 m^2^. The “Wuhan Main Urban Area Green Space System Plan (2011–2020)” (after this referred to as “the Plan”) proposes that by 2020, the planned indicator for per capita park green space in the urban core of Wuhan should reach 16.8 m^2^. In 2018, there were 10 park service units within the main urban area of Wuhan that met the standard requirements of “the Plan.” These parks were mainly located in Wuchang and Hongshan Districts, with the Shahu Park and the East Lake Scenic Area assuming the leading roles. Seven service units met the requirements of the “the Standards” but were lower than those of “the Plan.” These units were primarily situated in the northeastern tip of the study area. The distribution of a larger number of urban parks of a certain size and with fewer inhabitants are the main reasons for their exceptional accessibility.

##### 3.2.1.3 Per capita park size

**Figure 7:**
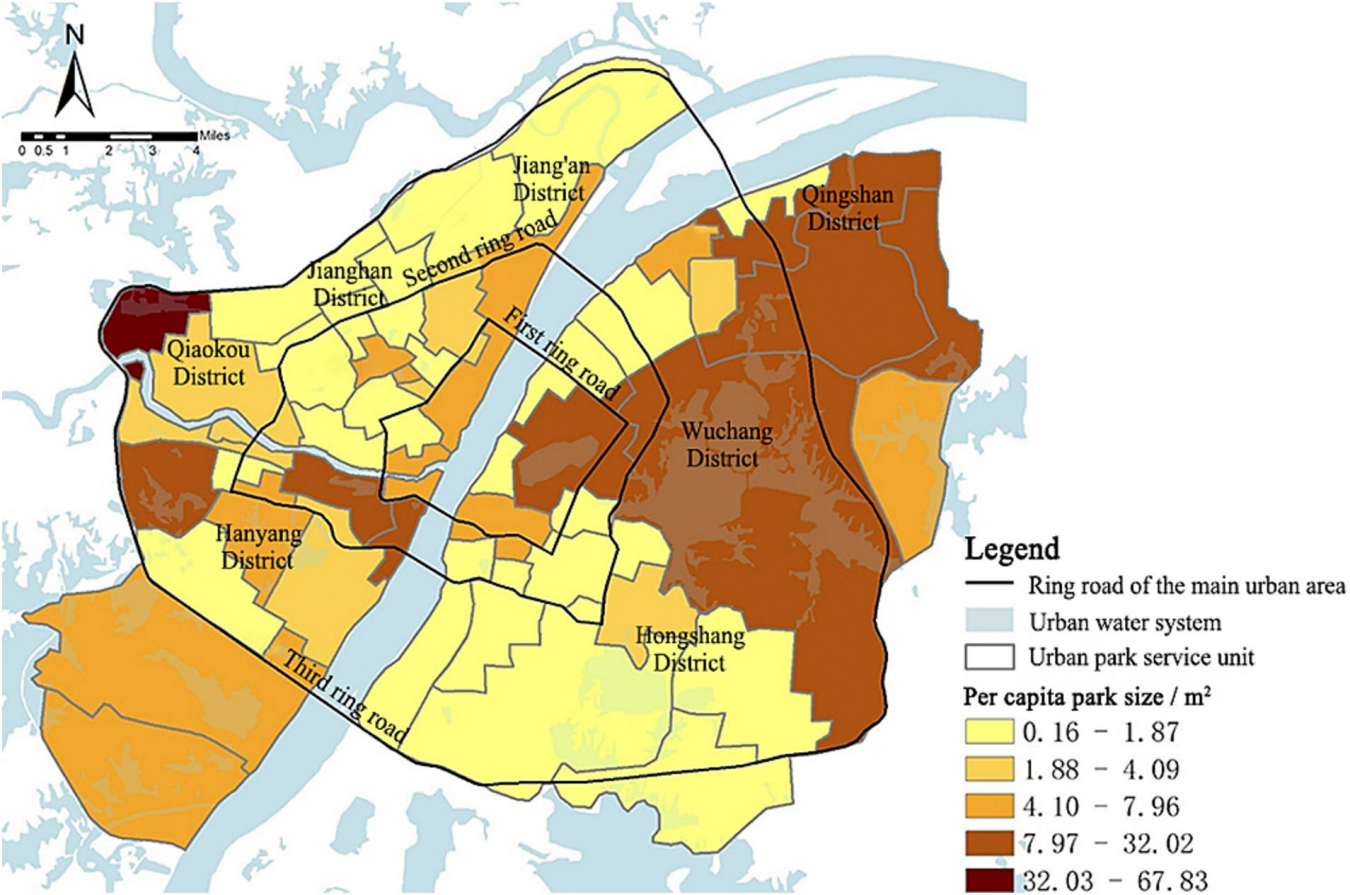
Per capita park size in each park service unit in the main urban area of Wuhan.

Figure 7 shows the distribution pattern of per capita park size in the study area. East Lake Scenic Area, Yangchun Lake, Qingshan Park, White Jade Park, and Bamboo Leaf Sea units had high per capita park areas, all of which reached 32.03 m^2^ or more. There were 25 service units with per capita park area lower than 1.87 m^2^, accounting for 52.08% of the total number of service units. The results indicate that some parts of the city experience an overwhelmed park supply capacity, resulting in a shortage in per capita park size. Hence, many residents in these underserved area still lack access to parks (52).

#### 3.2.2 Overall characterization of the park cold island supply capacity

**Figure 8:**
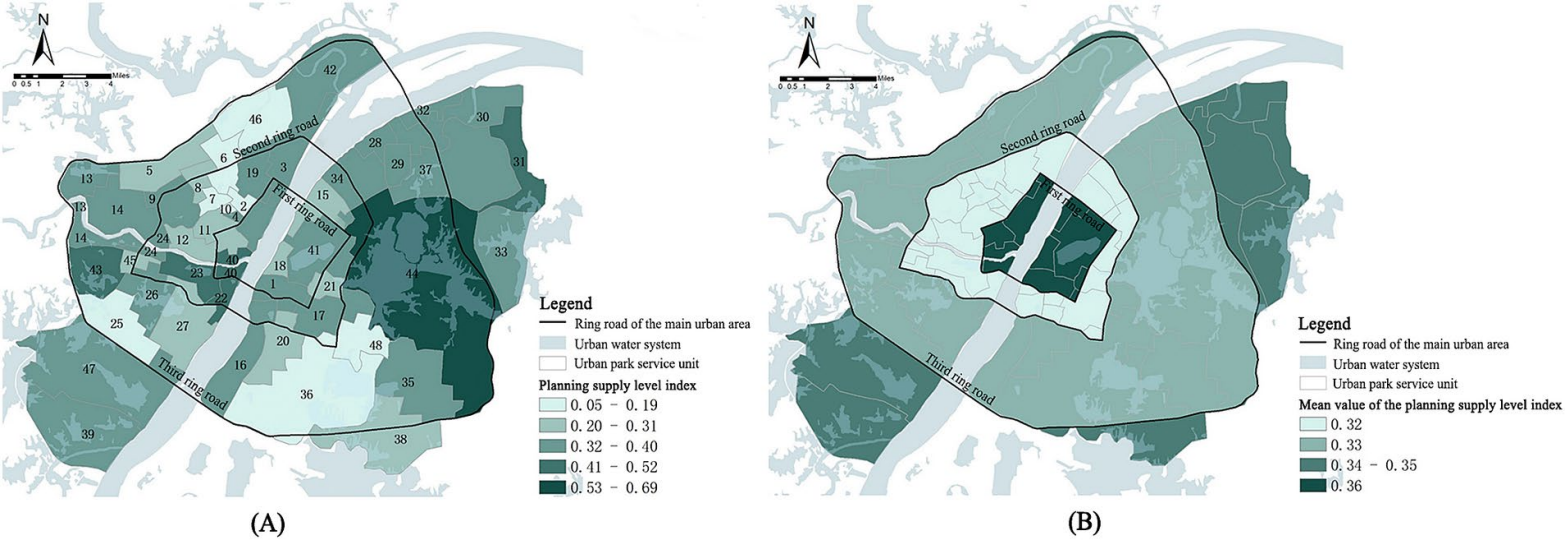
**(A)** Spatial distribution of the cold island supply capacity; **(B)** The cold island supply capacity within each ring road in the study area.

The calculated park cold island supply index SI was assigned to the corresponding spatial location. Then the spatial distribution map of the cold island supply capacity of urban parks in the main urban area of Wuhan was obtained (Figure 8A). By analyzing the map, the following characteristics were presented: ① The supply index of various units within the study area ranges from 0.05 to 0.69, demonstrating significant spatial heterogeneity. High-supply-capacity units are primarily concentrated around urban water systems, with the East Lake Scenic Area unit exhibiting the highest supply index, ranging from 0.53 to 0.69. In contrast, low-value areas are predominantly located in regions with lower vegetation coverage, such as the New District Park unit, South Lake unit, Houhu unit, and Zhuodaoquan Park unit within the second-third ring road of the study area, where the supply index is only 0.05–0.19. ② Furthermore, the average supply capacity of cold islands across different layers ranges from 0.32 to 0.36, indicating relatively minor overall variation (as shown in Figure 8B). The highest average cooling island supply capacity is observed within the first ring, followed by a gradual decrease and subsequent increase as the ring layers expand. Within the first-second ring road, the development and construction intensity are high, resulting in limited available space for park construction. Consequently, the cold island supply capacity is lower than that of other rings. Inside the second-third ring road are large areal lakes and parks, such as East Lake Scenic Area, Moon Lake Scenic Area, Wuhan Zoo, Ink Lake, etc. Despite that the intensity of urban construction is similar to that of the second ring road, a relatively high cold island supply is still maintained. Outside the third ring road, because of its proximity to the city’s outskirts, the intensity of development is diminishing. Consequently, there is more space to maintain its natural ecology in its original state, and the supply capacity of cold islands has been improved compared with that of the second ring road.

The findings of this study corroborate existing research on the critical role of water bodies in amplifying park cooling effects, as high-supply units typically feature a higher proportion of internal or adjacent water bodies. Notably, water bodies exhibit higher cooling intensity and magnitude compared to tree-dominated green spaces (53–55), particularly when exceeding 30% coverage within parks (28, 56). Furthermore, the synergistic interaction of blue-green spaces enhances cooling ranges and spatial impacts: water bodies improve thermal convection efficiency, while vegetation regulates radiative balance through shading and air circulation, collectively accelerating urban convection systems to establish localized cooling zones that surpass the performance of isolated landscape elements (57, 58). These mechanisms align precisely with the observed spatial distribution patterns, solidifying the robustness of our conclusions.

### 3.3 Evaluation of the residential cold island demand index from the demand perspective

#### 3.3.1 Visual analysis of residential cold island demand

##### 3.3.1.1 Population density

**Figure 9:**
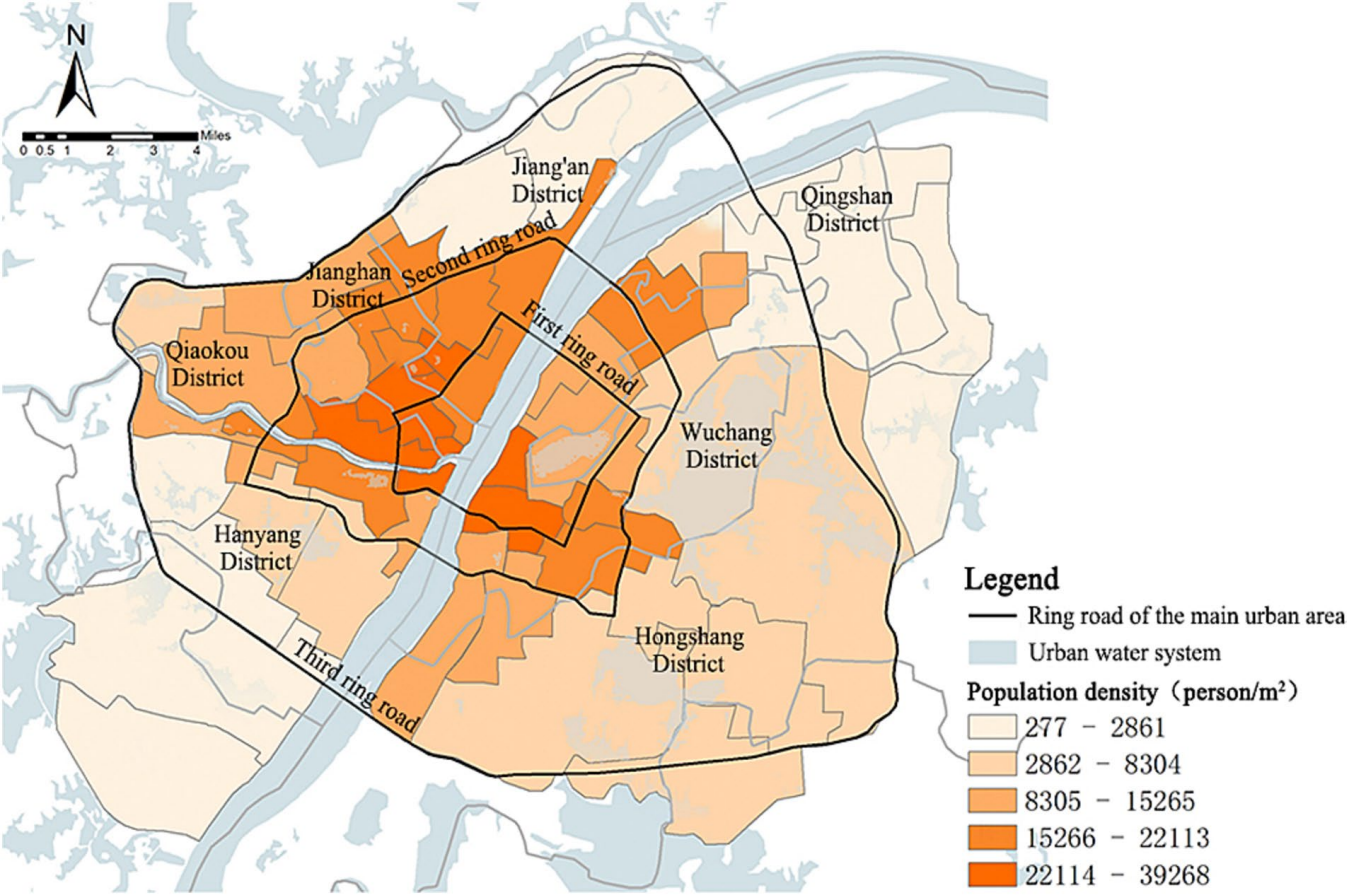
Population density distribution in the main urban area of Wuhan.

Figure 9 illustrates that the population density in the main urban area of Wuhan shows an overall trend of decreasing distribution with the Yangtze River serving as the central axis extending to the east and west wings. There were 8 high population density areas (PD > 8,000 people/km^2^), accounting for 16.67% of the total number of service units. These areas were mainly distributed in service units such as Yellow Crane Tower, Neisha Lake, Turtle Mountain Scenic Area, Zhongshan Park, Qiaokou Park, and Treasure Island Park, located at the confluence of the Yangtze River and the Han River. There were 10 low-value zones (PD < 2,861 people/km^2^), accounting for 20.83% of the total number of service units. These zones were concentrated in the northeastern, northwestern, and southwestern parts of the region, including Qingshan Park, White Jade Park, Yangchun Lake Park, Dijiao Park, Tang Lake Park, and other service units.

##### 3.3.1.2 POI kernel density

**Figure 10:**
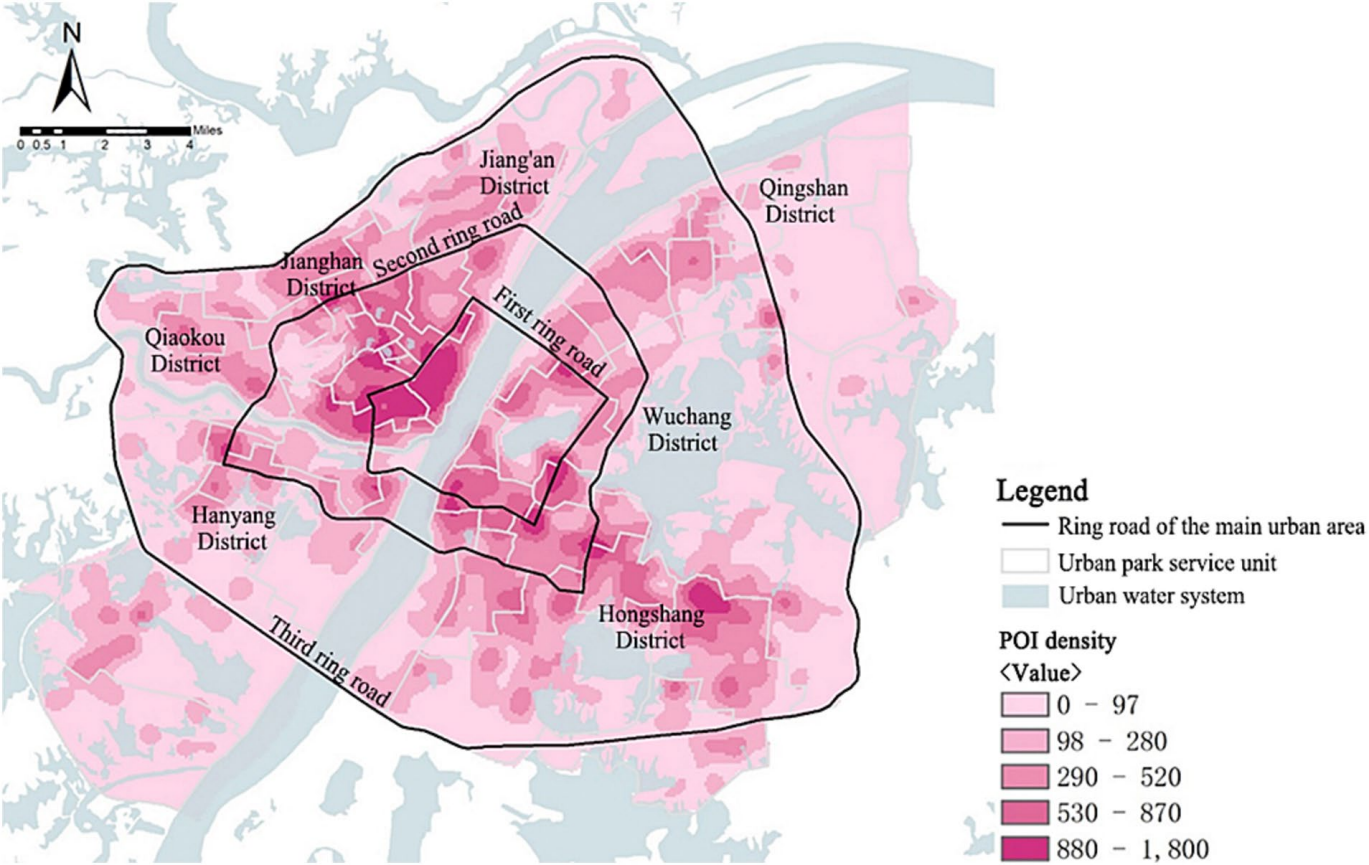
POI kernel density in the main urban area of Wuhan.

The kernel density of social activity POIs exhibited a geographical distribution pattern that aligned with the kernel density of the population (Figure 10). Within the first ring road is the core area of urban activities, and the POI density gradually decreased from the first ring road to the outside. The high-value areas (PoiKD >880) were mainly located in service units such as Zhongshan Park, Minor South Lake Park, Yellow Crane Tower, and Simei Tang Park along the banks of the river in the central region, as well as in the service units such as Guanshan Park, Ziyang Park, and Hongshan Park in the eastern Hongshan District. While the low-value areas (PoiKD <98) were centrally located in the outer part of the study area in the service units of White Jade Park, Yangchun Lake Park, Tang Lake, and Dijiao Park.

##### 3.3.1.3 Land surface temperature

**Figure 11:**
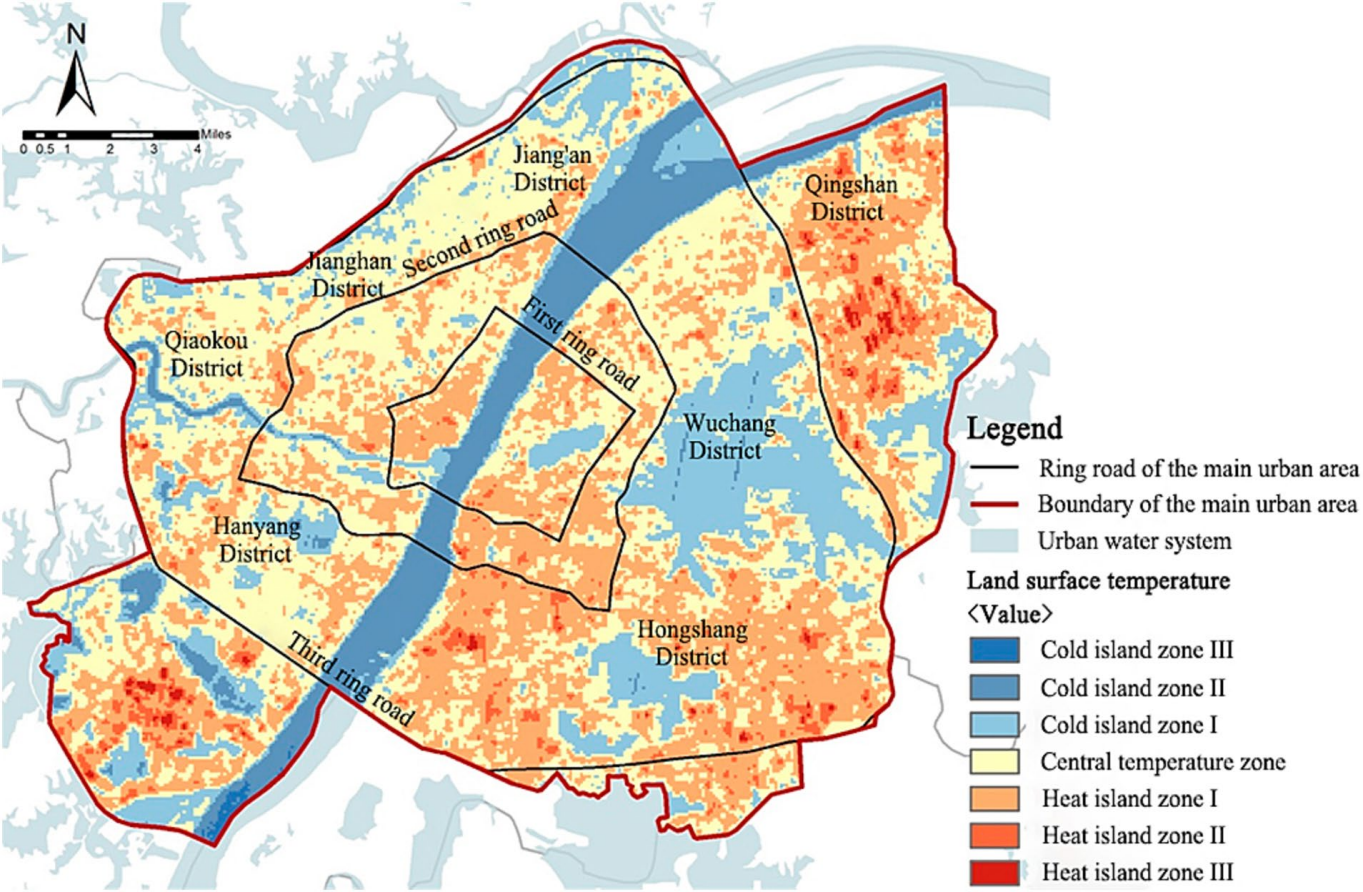
Inversion of land surface temperature in the main urban area of Wuhan.

The results of land surface temperature retrieval indicated that the land surface temperature in the main urban area of Wuhan ranged from 23.5°C to 56.1°, highlighting a significant temperature difference within the region (Table 4). The cold and heat islands in the study area exhibited a mosaic distribution pattern, with the cold island space mostly showing point and line distribution, while the heat island space shows a large and widely distributed pattern (Figure 11). Specifically, units adjacent to water bodies or containing large water areas have lower average temperatures, such as East Lake Scenic Area, the Yangtze River, Han River, South Prince Edward Lake, Longyang Lake, and Ink Lake, suggesting that large water bodies have a significant mitigating effect on high-temperature environments. The average temperatures within park service units at the northeastern and southeastern ends of the study area were higher, indicating a high risk of thermal environment and a high demand for cold islands among residents.

##### 3.3.1.4 Heat island patch size

**Figure 12:**
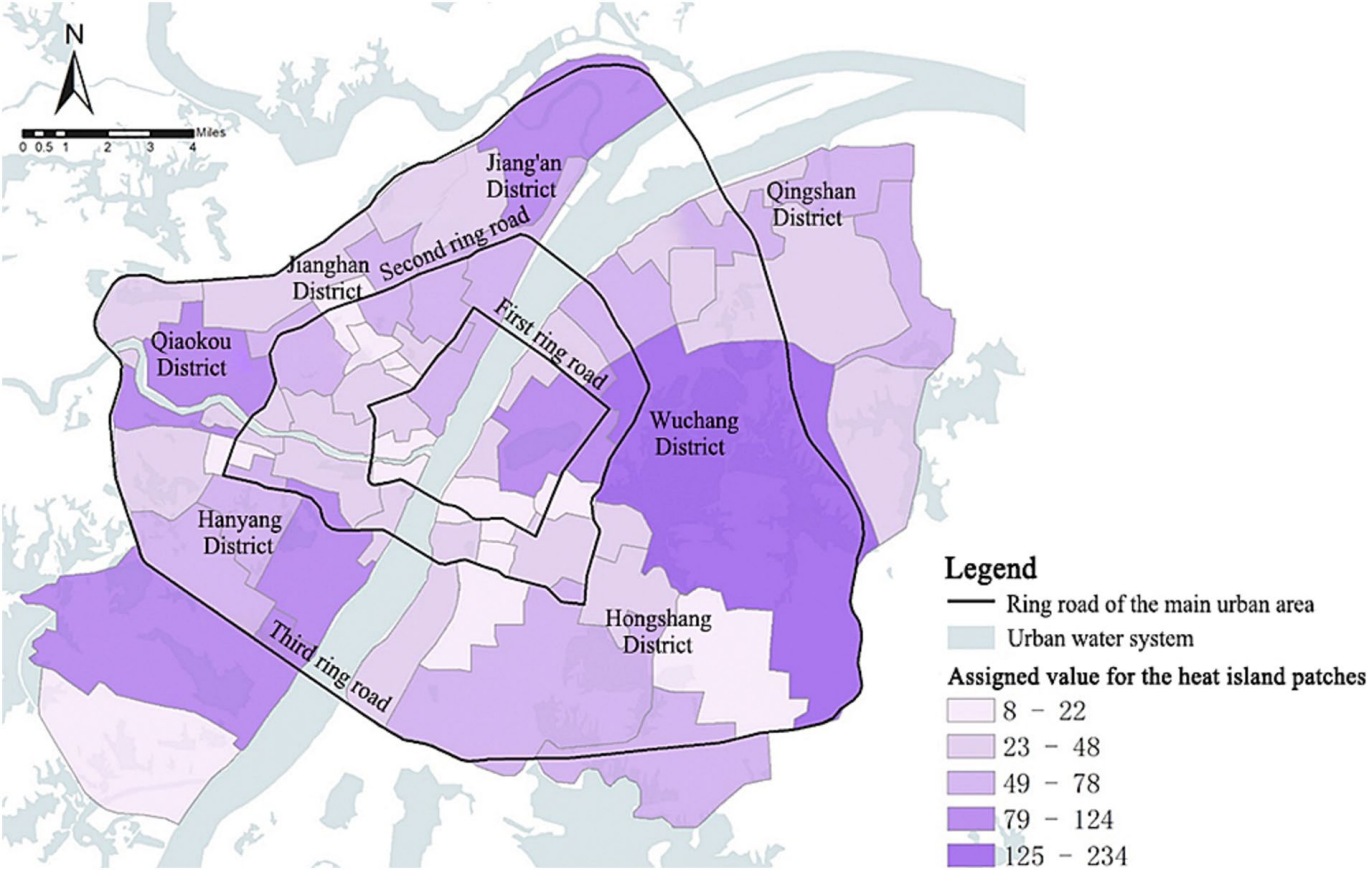
Spatial distribution of assigned values for heat island patches in the main urban area of Wuhan.

The score distribution map of heat island patches was obtained by counting the size of each heat island patch within each park service unit and assigning the corresponding value (Figure 12). The unit with the highest heat island patch score was No. 40 East Lake Scenic Area unit, with 24.6% of the range covered by heat islands. Among it, the largest heat island patch had a size of about 1,100 hm^2^, located in the East Lake High-tech Development Zone (Optics Valley). It suggests that the cold island effect of the East Lake Scenic Area exerts a limited influence, while the active development of the high-tech zone also generates significant heat. The unit with the highest percentage of heat island coverage was the No. 20 Chuwangtai unit, at 83.8 percent. As the unit is located along Baishazhou Avenue, the development intensity is high. This unit faces a challenge in terms of the scarcity of expansive, environmentally sustainable parks and green spaces within its boundaries. Consequently, it encounters difficulties in mitigating the effects of the urban heat island phenomenon, particularly during the summer season.

#### 3.3.2 Overall characterization of the residential cold island demand index

**Figure 13:**
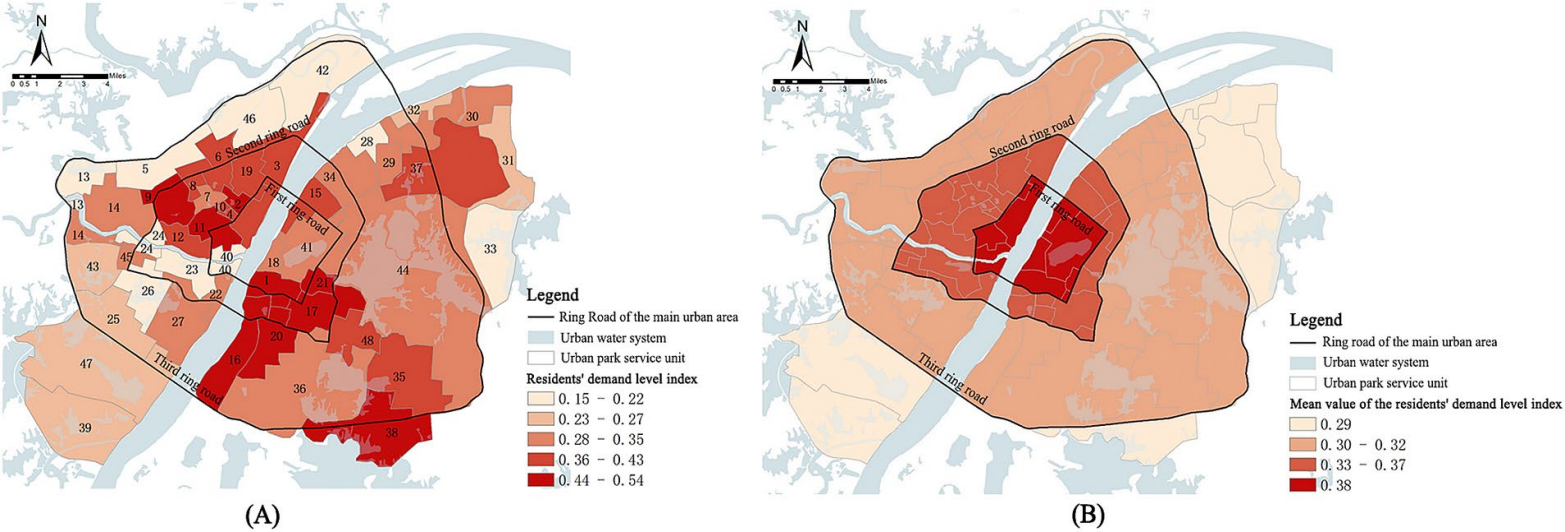
**(A)** Spatial distribution of the cold island demand index; **(B)** The cold island demand capacity within each ring road in the study area.

The calculated residents’ cold island demand index DI was assigned to the corresponding spatial location. Then the spatial distribution map of the residents’ cold island demand index in urban park units in the main urban area of Wuhan was obtained (Figure 13A). The darker the color of the unit color block, the stronger the demand for cold islands for residents. The following characteristics were presented: ① The demand level for cold islands in urban parks within the main urban area of Wuhan exhibits a spatial distribution characterized by “scattered across the entire region with localized concentrations.” The high-demand units match with the center of distribution of population density and POI density. Seven units, including Ziyang Park Unit, Hongshan Park Unit, Chuwangtai Unit, Hongshan Square Unit, Rhyme Lake Unit, Wangjiadun Unit, and Zhongshan Park Unit, had residential demand indexes ranging from 0.44 to 0.54, indicating a higher level of urgency in terms of resident demand. ② The average demand index is spatially linked to the first, second, and third ring circles of the main urban area of Wuhan (Figure 13B). The demand values of each circle decreased from the core area outwards. This suggests that in general, the demand-side indicators have a certain correlation with the intensity of urban development and construction. Furthermore, the first ring of Wuhan’s main urban area carries more functions of urban activities and attracts a large number of people to gather, resulting in elevated cooling island demand.

This finding aligns with Xin Ruhong et al.’s research on urban thermal regulation service demand, where communities with higher resident population density and POI density tend to face higher thermal environment risks, and thus have a higher level of cold island demand (59). The spatial distribution of population density and POI density, as an indirect characterization of the intensity of socio-economic activities, is closely related to the level of cold island demand. Studies have shown that anthropogenic heat generated by high-intensity socio-economic activities significantly raises surface and air temperatures in the region and surrounding (60). In areas with high urban development intensity, on the one hand, the rise in the proportion of impervious surfaces leads to a reduction in heat capacity, exacerbating the heat island effect and elevating the surface base temperature (61–63); on the other hand, dense high-rise buildings seriously impede air circulation and inhibit the spread of the cold island effect (64, 65). Therefore, the correlation mechanism between urban development intensity and the level of cold island demand is mainly reflected in the expansion of impervious surfaces and the deterioration of ventilation conditions, which provides scientific support for the regulation of urban thermal environment and the optimization of park planning.

### 3.4 Identification and evaluation of supply and demand balance relationship of cold islands in urban parks

**Figure 14:**
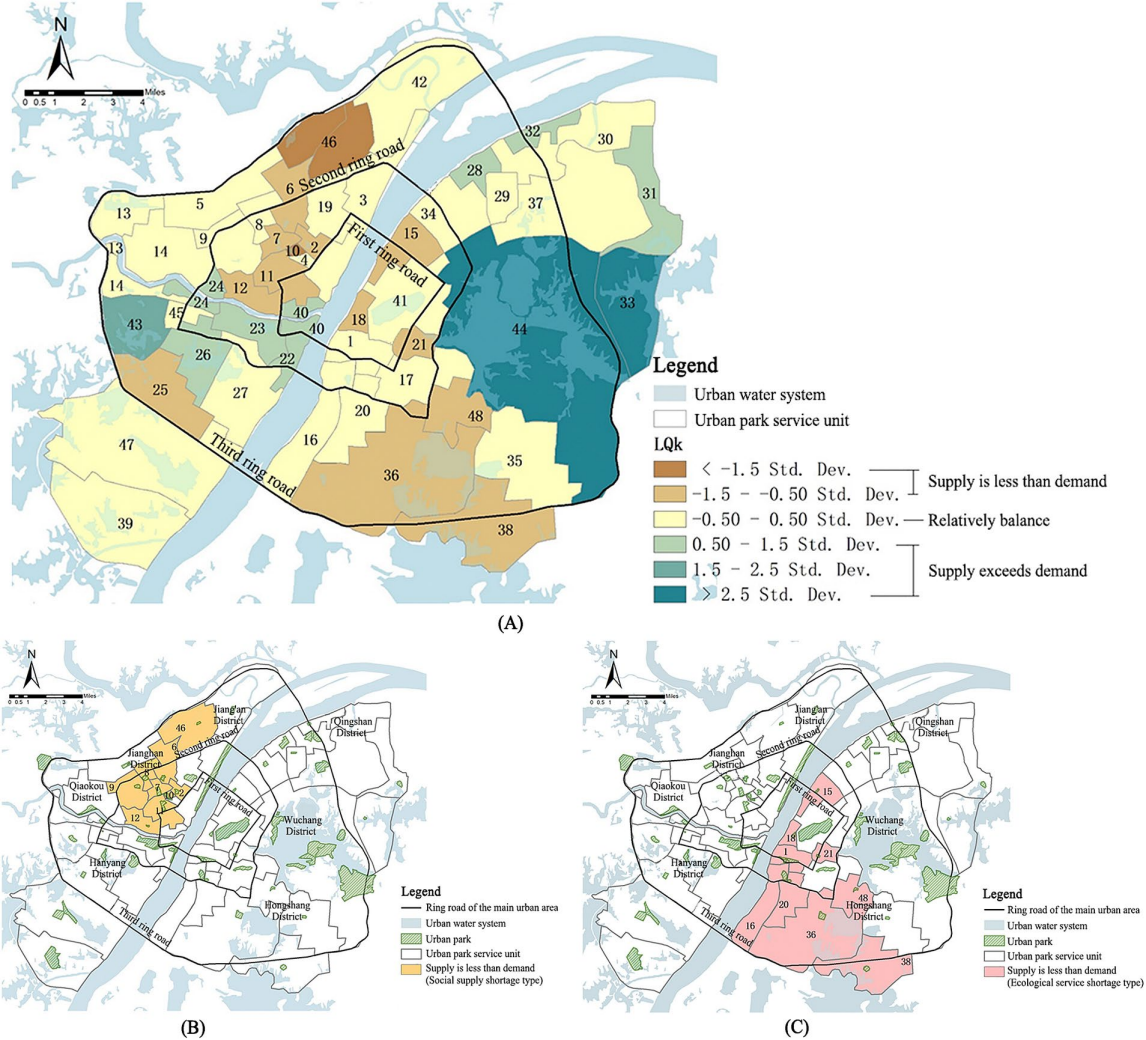
**(A)** Spatial distribution of the supply–demand relationship of cold island effect in the main urban area of Wuhan; **(B)** Spatial distribution of the social supply-shortage type units; **(C)** Spatial distribution of the ecological service-shortage type units.

From the park cold island supply indicator (SI) and resident cold island demand indicator (DI) of the 48 park service units in the study area, the location entropy theory was introduced to evaluate the level of balance between supply and demand. The spatial distribution map of the cold island effect supply and demand was obtained by connecting the supply and demand relationship with the spatial location of the units (Figure 14A).

#### 3.4.1 Supply and demand I: less supply but more demand

There are 19 units in total, showing a spatially clustered distribution in the study area, belonging to regions with high residential density and lacking vegetation cover. Two types are analyzed: the social supply-shortage type (Figure 14B) and the ecological service-shortage type (Figure 14C).

Social supply-shortage type (9 units): The main manifestations of this type lie in the residents’ impeded access to the cold island effect, the excessive people cold island supply of urban parks carries, and the not sufficiently complementary built environment around the dominant parks in the unit to the cold island supply. These regions are in the middle of Qiaokou District, Jianghan District, and Jiang’an District. The confluence of the Yangtze River and the Han River has facilitated the gradual development of this area into the commercial and financial center of Wuhan, and a heavily populated zone in the main urban area, which mainly explains its high social demand value. Simultaneously, the existing neighborhoods exhibit a high level of density, resulting in “numerous but small parks” in the region, hence presenting the overloaded supply capacity phenomenon.

Ecological service-shortage type (10 units): The cold island supply of urban parks within the units is insufficient to mitigate the summer heat and the heat island effect caused by urbanization, resulting in extreme heat. Additionally, the continuous distribution of high-temperature heat island patches in some units exacerbates the negative impacts of the heat island effect. These regions are located in Hongshan District and Wuchang District, which include rapidly developing zones such as the East Lake High-Tech Development Zone, Hongshan Square, Zhongnan Road Business District, and Optics Valley University Town. The parks in these units are small and dispersed, resulting in a very weak cooling effect on land surface temperature. Although adjacent to three water bodies-East Lake, South Lake, and the Yangtze River, the connectivity among them is weak, resulting in inadequate dispersion of the cold island effect. This demonstrates that, in addition to park size, landscape connectivity is also a critical factor influencing cold island supply. This finding aligns with previous research, confirming that the mosaic distribution of cold and heat islands and the connectivity of cold island networks play a crucial role in improving thermal environmental issues. A spatially complete and well-structured cooling network can significantly reduce the continuity of negative impacts associated with high-intensity urban development (66–68).

#### 3.4.2 Supply and demand II: relative balance between supply and demand

There are 18 units in total (Figure 14A). These units exhibit a balanced relationship between park cold island supply and residential cold island demand within a specific range. However, given the constant development of the city and the corresponding changes in residents’ demands, it is imperative to analyze these units in conjunction with the urban land use plan in order to detect any difficulties possibly requiring early warning.

#### 3.4.3 Supply and demand III: more supply but less demand

There are 11 units in total (Figure 14A), each exhibiting a park vegetation coverage of 0.75 or higher. Additionally, all of these units either contain water bodies or are situated in close proximity to them. The supply capacity of cold islands is strong enough to meet the residents’ demand for cold islands within the unit. For example, the East Lake Scenic Area, Longyang Lake-Ink Lake Scenic Area, Moon Lake Scenic Area, Fuhe Ecological Green Wedge, Hanjiang River Ecological Axis, etc., collectively form the ecological framework of the main urban area of Wuhan and designated in the planning as the low development intensity or ecological landscape control zones, which is conducive to the sustainable development of the city and the healthy life of its inhabitants “benign balance.

## 4 Discussion

### 4.1 Urban park planning strategy for matching supply and demand of cold islands

#### 4.1.1 Overall strategy–adaptive planning

**Figure 15:**
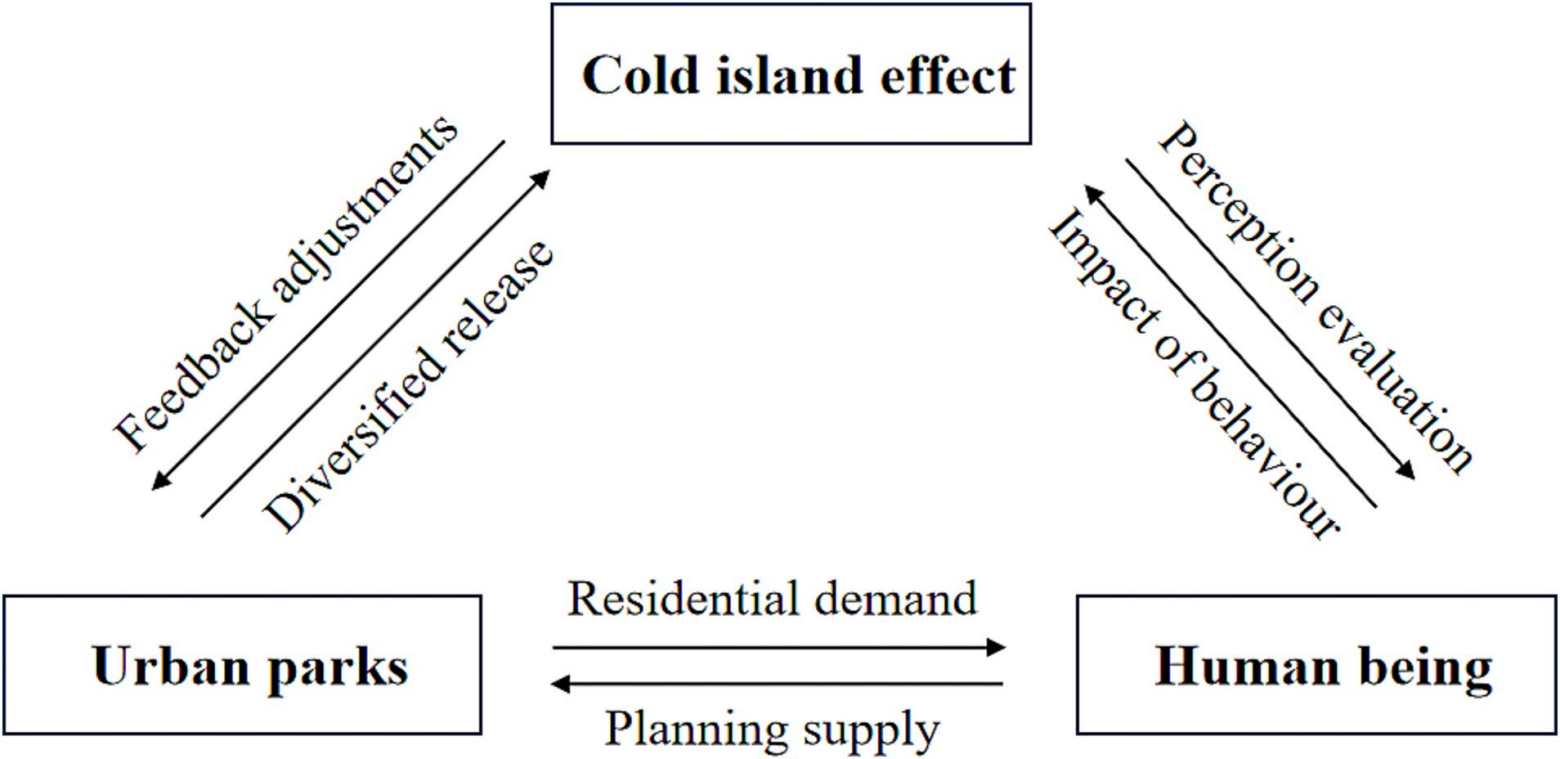
Operational mechanism of adaptive planning structures.

Adaptive planning is a spatial feedback process aiming to dynamically adjust planning strategies in response to the evolving requirements of the users (69). To ensure a relative equilibrium between the supply and demand of cold islands in urban parks in the future, it is imperative to introduce the concept of adaptive planning and conduct prognostic assessments. The operational mechanism of the adaptive planning structure employed in this study is visually depicted in Figure 15. It analyses the intrinsic linkage between urban parks, cold island effects, and people. This analysis is to reveal the problems of the current space in terms of supply and demand and to assess the development potential of the future space. It can guide the adaptive planning process of urban parks, the object of adaptation, and characterize the dynamic demand changes of urban residents, the subject of adaptation (70).

#### 4.1.2 Typology planning strategy

Taking the evaluated supply and demand relationship as the guide for action, this study presents a set of sub-strategy that exhibit strong feasibility and serve as valuable references. These strategies aim to enhance the cold island supply level of urban parks, bolster their adaptive capacity in the future development process and allocate the limited ecological resources and service functions to the individuals and regions that require them the most.

Strategy 1: During the process of old city reconstruction, it is advisable to plan for wedge-shaped parks and expand the existing parks, thereby enhancing the accessibility to urban parks (for less supply but more demand--social supply-shortage type). Currently, Wuhan is continuously promoting old city reconstruction and gradually and piecewise carrying out scientific planning. Emphasis should be placed on incorporating the consideration of the parks’ cold island effect into the renewal planning for Hankou, Qiaokou, and Jiang’an Districts. Liudu Bridge and Unity Street can consider planning city wedge parks to connect with the Yangtze River, when carrying out old city reconstruction. Some neighborhoods in Hanzhong Street, the Friendship Neighborhood, or other neighboring areas can consider connecting with Qiaokou Park to expand the size of Qiaokou Park when undergoing renovation and renewal. Moreover, they should reserve sufficient space for the expansion of city parks to meet the future demand for cold islands in the high-density residential neighborhoods. By controlling the building density and architectural form of old renovation communities, and using more point buildings at air intakes, it can promote breeze circulation and effectively form their own fresh air systems. Through the accessibility analysis, the urban-facing joints and pedestrian entrances are increased to achieve the goal that city parks are accessible to more urban residents. For example, Northwest Lake Park, Zhongshan Park, Fountain Park, Minor South Lake Park, Treasure Island Park, Wuhan Youth Palace, and other parks and green spaces are relatively close to each other and they can consider planning the “string of pearls into a chain” of greenways. Planning greenways from residential neighborhoods to parks allows residents to reach urban parks more conveniently, improving the comfort and accessibility of paths leading to the parks. In the old city renovation, green space should be inserted in gaps or white space, and “pocket parks” with recreational and ornamental functions should be established to cover greenfield cold island service blind spots and enhance the cooling radiation effect on the neighborhood.

Strategy 2: Cold island connectivity should be enhanced and blue-green cold island corridors should be built (for less supply but more demand--ecological service-shortage type). Numerous studies have shown that cold island connectivity can improve cooling efficiency. From the southern suburbs of Wuhan along Tangxun Lake, South Lake, and East Lake to Tianxing Sandbar, a cold island corridor running through the north and south of Wuhan is constructed, passing through many heat island areas in the Wuchang and Hongshan Districts. It can cut down on the negative impacts of the heat island effect and alleviate the physical and mental discomfort due to the high temperature in summer. East Lake, South Lake, and Yezhi Lake are several essential waters in Wuchang District. They should reserve the open green space around the waters, enrich the vegetation community level, and play the synergistic cooling effect of the blue-green space to boost the overall cooling of the city.

Strategy 3: The blue-green intertwined ecological background should be restored and protected (for relative balance). It’s imperative to restore and protect the blue and green natural ecological background of the parks and to control and prevent the trend of encroachment on green spaces and water areas during urbanization. The blue-green intertwined urban parks, such as Tang Lake Unit, Sand Lake Unit, Yangchun Lake Unit, Wuhan Zoo Unit, etc., should optimize the edge shape of the blue-green space patches and keep the waterfront space with a high degree of vegetation coverage. It can not only better realize the cooling effect of the parks’ cold islands, but also provide citizens with better social and sports space thus to cope with the inevitable increase in the demand value of the cold islands in the process of urban development and to maintain the balance of supply and demand as long as possible.

Strategy4: Adequate green space should be reserved (for relative balance). Based on the early warning problems of each research unit in the relative balance zone, combined with Wuhan’s territorial spatial planning, the planning early warning strategy for the disorder of supply and demand in the development process are targeted to be proposed. The units with pieces of residential neighborhoods in the future plan, such as Jiangtan Sports Unit, Science Park Unit, Guanshan Unit, etc., should pay more attention to the budget for the number of potential residents, and analyze the accessibility of parks’ sites in the planning of urban parks. These units also are required to design community-level parks as a supplement and reserve a sufficient amount of land for green space development. This will prevent future urban area development from attracting too many residents and excessive social demand arising from socio-economic activities which will upset the existing relative balance, and enhance accurate prevention and control capability of urban thermal environmental risks.

### 4.2 Limitations and insights

This study represents a positive attempt to investigate the cold island effect of urban parks from a dual perspective of supply and demand. However, there are still challenges that require resolution. First of all, the cold island effect of urban parks exhibits significant spatiotemporal complexity; however, due to the inherent limitations of Landsat data, existing research predominantly focused on spatial analysis. Future studies can further investigate daily and seasonal variations in the supply and demand of the urban parks’ cold island effect by utilizing data sources with shorter revisit periods, such as MODIS (Moderate Resolution Imaging Spectroradiometer). This would help uncover the interaction mechanisms between park characteristics and meteorological factors across multiple temporal scales. Secondly, the supply and demand of the park cold island effect are influenced by multiple factors, such as internal landscape composition (71) and surrounding urban morphology (18), which were not comprehensively incorporated in the current evaluation system. Additionally, the selected indicators primarily explored urban thermal environment patterns at a two-dimensional level, lacking investigation into three-dimensional factors such as building height (BH), sky view factor (SVF), and frontal area index (FAI) (18). Future research should establish a more holistic evaluation framework for supply and demand to better analyze the complex environmental interactions surrounding urban parks. Finally, with the development of information technology, further investigation of urban parks can be conducted at a more detailed level, which should incorporate the integration of microscopic, mesoscopic, and macroscopic scales. This entails grasping the overall layout at the macroscopic scale, emphasizing the precise adjustments made at the micro level and considering the transitional function of the mesoscopic level. Future research can examine each unit on the grid scale to find the implementation points more precisely.

## 5 Conclusion

In the process of rapid urban development, the planning and construction of urban parks are prone to be a mismatch between supply and demand. In light of this, this paper selected 60 urban parks in the main urban area of Wuhan that effectively exert the cold island effect as the research subjects. By integrating the Thiessen polygons with the detailed regulatory planning units to delineate research units and developing the supply and demand evaluation model of the cold island effect of urban parks, this research conducted a comprehensive quantitative assessment of cooling services from both supply and demand perspectives. The interaction between the supply and demand perspectives was established by applying the location entropy theory, enabling the proposal of targeted optimization strategy for urban parks in Wuhan to achieve better supply–demand alignment. The main findings are as follows: ① The spatial distribution of cold island supply and demand exhibited significant heterogeneity. High-supply units were strongly correlated with water body distribution, while high-demand units aligned closely with population density and POI density centers, displaying a “scattered overall, locally concentrated” pattern. ② A significant supply–demand mismatch in cold island effects was observed, with 19 units (accounting for approximately 40%) exhibiting insufficient supply relative to demand. These units were predominantly concentrated in areas with complex building environments, high population density, low vegetation coverage, and poor landscape connectivity. To address these challenges, this study proposed the following optimization strategy: optimizing the scale and layout of existing parks, reserving green spaces for ecological restoration, strengthening the protection of blue-green ecological foundations, and establishing a blue-green cold island corridor network to enhance ecological connectivity.

By quantifying the residential cold island demand and the park cold island supply capacity, this study advances research on the cold island effect of urban parks at regional and city scales, offering new insights for sustainable urban development and climate adaptation planning. The findings not only provide a scientific basis for mitigating urban heat island effects but also offer decision-making support for the precise allocation of urban park resources. This research holds significant practical implications for improving urban living environments and enhancing ecological benefits with precision.

## Data Availability

The original contributions presented in the study are included in the article/Supplementary material, further inquiries can be directed to the corresponding author/s.

## Footnotes

^1^ http://glovis.usgs.gov/

^2^data source: https://www.openstreetmap.org/, acquired in 2018

^3^data source: https://lbs.amap.com/, accessed in 2018

^4^data source: https://www.worldpop.org/, accessed in 2018
