# Supplementary material for: Evaluation of the cold island effect of the urban parks in the main urban area of Wuhan from the perspective of supply and demand
Source: Front Public Health. 2025 Mar 17;13:1523210. doi: 10.3389/fpubh.2025.1523210 (PMC11955679; doi:10.3389/fpubh.2025.1523210)
Supplement: Supplementary file 1 [file Table_1.docx]

Supplementary Material

Evaluation of the Cold Island Effect of the Urban Parks in the Main Urban Area of Wuhan from the Perspective of Supply and Demand

Jufang Song^1*^, Yongxuan Qiao^1^, Yihan Liu^1^

^1^School of Urban Design, Wuhan University, Wuhan, China

*** Correspondence:** Jufang Song^*^ [sjufang@whu.edu.cn](mailto:sjufang@whu.edu.cn)

# Supplementary Figures and Tables

## Supplementary Tables

**Supplementary Table 1.** Park Service Unit Information.

| **Unit number** | **Unit name** | **Name of the dominant park** | **Unit size/km²** |
| --- | --- | --- | --- |
| 1 | Yellow Crane Tower  Park Unit | Yellow Crane Tower Park | 6.17 |
|  |  | Shouyi Park |  |
|  |  | Xinhai Memorial Square |  |
| 2 | Treasure Island Park Unit | Treasure Island Park | 1.95 |
| 3 | Hankou River Beach Unit | Hankou River Beach Park | 14.94 |
| 4 | Minor South Lake Unit | Minor South Lake Park | 0.64 |
| 5 | Evergreen Park Unit | Evergreen Park | 11.58 |
| 6 | Lingjiao Lake Unit | Lingjiao Lake Park | 7.07 |
| 7 | Northwest Lake Unit | Northwest Lake Greening Square | 2.30 |
| 8 | Houxiang River Unit | Houxiang River Park | 3.60 |
| 9 | Wangjiadun Unit | Wangjiadun Park | 8.23 |
| 10 | Fountain Park Unit | Fountain Park | 0.51 |
| 11 | Zhongshan Park Unit | Zhongshan Park | 6.49 |
| 12 | Qiaokou Park Unit | Qiaokou Park | 4.84 |
| 13 | Bamboo Leaf Sea Park Unit | Bamboo Leaf Sea Park | 7.24 |
| 14 | River Beach Sports Unit | Rive Beach Sports Park | 17.20 |
| 15 | Simei Tang Unit | Simei Tang Park | 6.85 |
| 16 | Ziyang Park Unit | Ziyang Park | 13.61 |
| 17 | Hongshan Park Unit | Hongshan Park | 10.82 |
| 18 | Neisha Lake Unit | Neisha Lake | 2.47 |
| 19 | Liberation Park Unit | Liberation Park | 7.46 |
| 20 | Chuwangtai Unit | Chuwantai Ruins Park | 10.03 |
| 21 | Hongshan Square Unit | Hongshan Square | 3.31 |
| 22 | Hanyang River Beach Unit | Hanyang Lotus Lake Park | 3.07 |
|  |  | Hanyang River Beach Park |  |
| 23 | Moon Lake Unit | Qintai Greening Square | 6.69 |
|  |  | Moon Lake Scenic Area |  |
| 24 | Hanshui Park Unit | Hanshui Park | 3.50 |
| 25 | New District Park Unit | New District Park | 13.11 |
| 26 | Ink Lake Unit | Ink Lake Park | 9.90 |
| 27 | Wuhan Zoo Unit | Wuhan Zoo | 15.50 |
| 28 | Peace Park Unit | Peace Park | 3.70 |
| 29 | South Main Canal  Playground Unit | South Main Canal Playground | 4.34 |
| 30 | Qingshan Park Unit | Qingshan Park | 16.50 |
|  |  | Daijia Lake Park |  |
| 31 | White Jade Park Unit | White Jade Park | 12.03 |
| 32 | Tinxing Sandbar Bridge Unit | Tinxing Sandbar Bridge Park | 3.50 |
| 33 | Wudong Park Unit | Wudong Park | 18.23 |
| 34 | Science Popularization  Park Unit | Garden Science  Popularization Park | 10.80 |
| 35 | Guanshan Unit | Guanshan Dutch-Style Garden | 20.33 |
| 36 | South Lake Unit | South Lake Happy Bay Park | 43.99 |
| 37 | Yangchun Lake Unit | Yangchun Lake Park | 30.97 |
| 38 | Yunhu Unit | Yunhu Park | 22.58 |
| 39 | Tang Lake Unit | Tang Lake Park | 25.90 |
| 40 | Turtle Mountain Unit | Turtle Mountain Park | 3.42 |
| 41 | Sand Lake Unit | Sand Lake Park | 15.88 |
| 42 | Dijiao Park Unit | Dijiao Park | 22.58 |
| 43 | Shoulder Pole Hill Unit | Shoulder Pole Hill Cemetery | 10.60 |
| 44 | East Lake Scenic Area Unit | East Lake Chufeng Garden | 94.47 |
|  |  | East Lake Falling Geese  Scenic Spot |  |
|  |  | East Lake Plum Garden |  |
|  |  | East Lake Mill Hill Scenic Spot |  |
|  |  | East Lake Hearing Waves Scenic Spot |  |
|  |  | Fengdu Mountain Park |  |
|  |  | Ma'anshan Forest Park |  |
|  |  | Mill Hill Botanical Garden |  |
| 45 | Futabashi Cultural  District Unit | Futabashi Cultural  District quare | 2.35 |
| 46 | Houhu Unit | Houhu Park | 14.39 |
| 47 | South Prince Edward  Lake Unit | South Prince Edward Lake  Sports Park | 42.42 |
| 48 | Zhuodaoquan Park Unit | Zhuodaoquan Park | 8.46 |
